# PIK-III exerts anti-fibrotic effects in activated fibroblasts by regulating p38 activation

**DOI:** 10.1371/journal.pone.0306624

**Published:** 2024-09-06

**Authors:** Santiago Sanchez, Aaron K. McDowell-Sanchez, Sharaz B. Al-Meerani, Juan D. Cala-Garcia, Alan R. Waich Cohen, Scott A. Ochsner, Neil J. McKenna, Lindsay J. Celada, Minghua Wu, Shervin Assassi, Ivan O. Rosas, Konstantin Tsoyi

**Affiliations:** 1 Section of Pulmonary, Critical Care, and Sleep Medicine, Department of Medicine, Baylor College of Medicine, Houston, TX, United States of America; 2 Department of Molecular and Cellular Biology, Baylor College of Medicine, Houston, TX, United States of America; 3 Division of Rheumatology, University of Texas Health Science Center at Houston, McGovern Medical School, Houston, TX, United States of America; Justus - Liebig University Giessen, GERMANY

## Abstract

Systemic sclerosis (SSc), also known as scleroderma, is an autoimmune-driven connective tissue disorder that results in fibrosis of the skin and internal organs such as the lung. Fibroblasts are known as the main effector cells involved in the progression of SSc through the induction of extracellular matrix (ECM) proteins and myofibroblast differentiation. Here, we demonstrate that 4’-(cyclopropylmethyl)-N2-4-pyridinyl-[4,5’-bipyrimidine]-2,2’-diamine (PIK-III), known as class III phosphatidylinositol 3-kinase (PIK3C3/VPS34) inhibitor, exerts potent antifibrotic effects in human dermal fibroblasts (HDFs) by attenuating transforming growth factor-beta 1 (TGF-β1)-induced ECM expression, cell contraction and myofibroblast differentiation. Unexpectedly, neither genetic silencing of PIK3C3 nor other PIK3C3 inhibitors (*e*.*g*., SAR405 and Autophinib) were able to mimic PIK-III-mediated antifibrotic effect in dermal fibroblasts, suggesting that PIK-III inhibits fibroblast activation through another signaling pathway. We identified that PIK-III effectively inhibits p38 activation in TGF-β1-stimulated dermal fibroblasts. Finally, PIK-III administration significantly attenuated dermal and lung fibrosis in bleomycin-injured mice.

## Introduction

Systemic Sclerosis (SSc) is an autoimmune disease with poorly understood etiology. The most frequently observed clinical manifestations of SSc include fibrosis of the skin and internal organs (e.g., lungs) [1]. SSc-associated interstitial lung disease (ILD), primarily pulmonary fibrosis, is the leading cause of death among SSc patients [2, 3]. Approximately 30% of SSc patients develop progressive SSc-ILD that contributes significantly to patient mortality [4–6]. However, despite high mortality and morbidity rates, therapies to treat commonly observed fibrotic complications of SSc are limited [7].

Fibroblasts belong to the main effector cells of SSc and pulmonary fibrosis [8]. In response to profibrotic factors such as TGF-β, fibroblasts undergo myofibroblast transformation, produce excessive ECM proteins and initiate contractile protein activation. Simultaneous activation of these factors was shown to promote tissue remodeling and fibrosis [9]. Thus, targeting these profibrotic responses in SSc fibroblasts is a potential therapeutic intervention for the treatment of SSc and SSc-ILD.

PIK-III is a small molecule inhibitor of PIK3C3, also known as vacuolar protein sorting 34 (VPS34), a class III phosphoinositide 3-kinase (PI3K) known to phosphorylate phosphatidylinositol to generate phosphatidylinositol 3-phosphate (PI(3)P) [10]. PIK-III exerts its function by regulating PIK3C3-dependent processes, such as autophagy and endosome trafficking [10]. Although PIK3C3 inhibition has proven beneficial in kidney fibrosis, the therapeutic effects of PIK-III in dermal or pulmonary fibrosis remain to be elucidated [11]. PIK3C3 deficiency was also shown to significantly reduce severe autoimmunity in models of experimental autoimmune encephalomyelitis [12–16]. Together, these studies suggest that PIK-III may be beneficial for the treatment of autoimmune-specific skin and pulmonary fibrosis, i.e., SSc and SSc-ILD. Here we found that PIK-III treatment effectively decreased dermal and lung fibrosis by attenuating fibroblast activation. Unexpectedly, both PIK3C3 silencing using shRNA and other PIK3C3 inhibitors were unable to mimic the PIK-III-mediated therapeutic effect observed in fibroblasts, suggesting that PIK-III exerts its function independently of VPS34. Using an antibody phosphorylation array, we identified p38 activation as a novel target of PIK-III. In accordance, p38 inhibition using SB203580 significantly attenuated profibrotic responses in dermal fibroblasts that resembled those observed after PIK-III treatment. We also observed that overexpression of p38 could reverse anti-fibrotic effect of PIK-III in activated fibroblasts. Thus, our findings provide a support for the use of the small molecule inhibitor PIK-III as a therapeutic option to regulate p38-mediated fibroblast activation in dermal and lung fibrosis.

## Results

### PIK-III inhibits TGF-β1-induced profibrotic responses in human dermal fibroblasts (HDFs)

**Fig 1:**
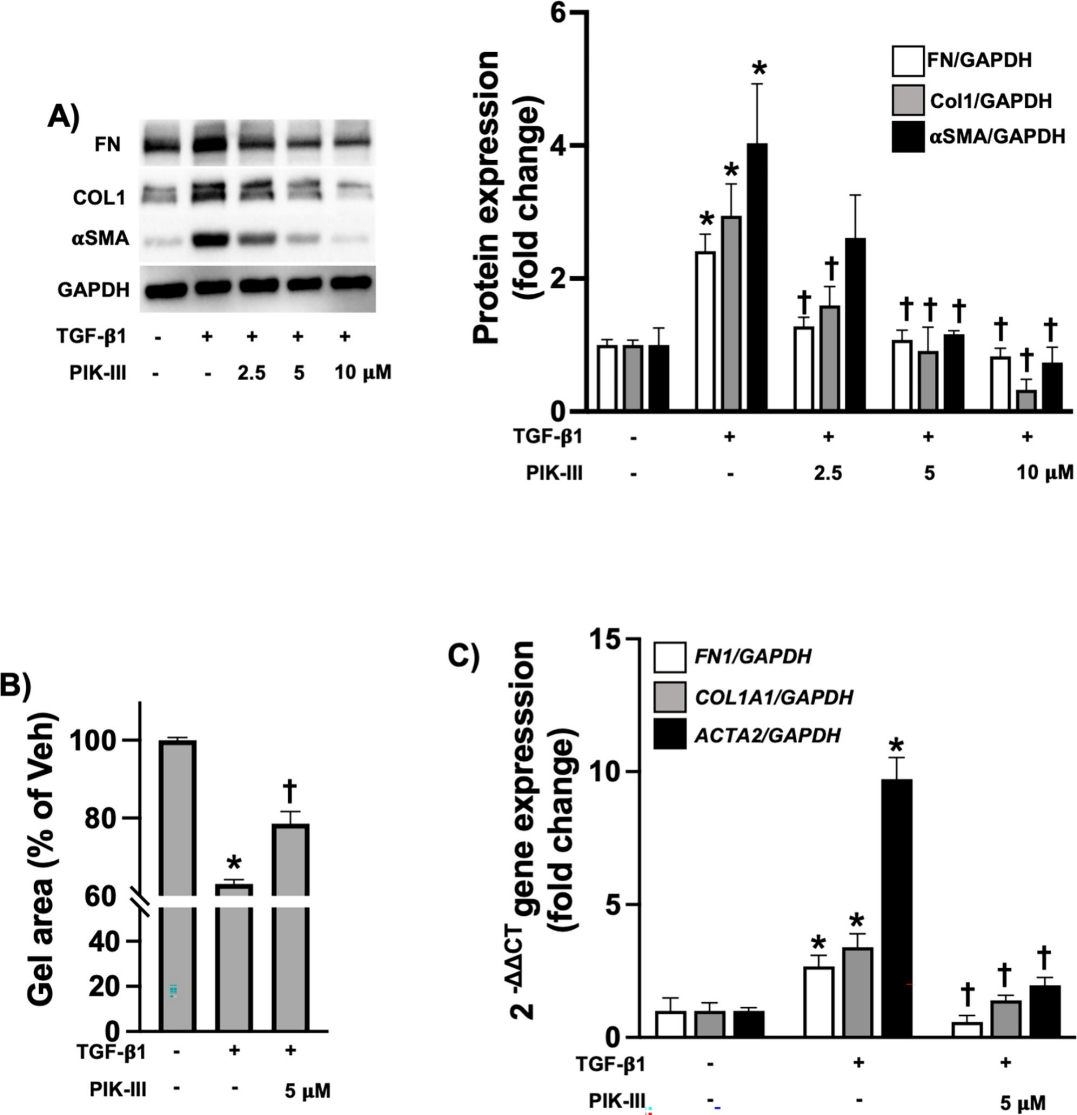
PIK-III attenuates ECM production and myofibroblast differentiation in activated HDFs. **A)** HDFs were stimulated with TGF-β1 (10 ng/ml) in the presence or absence of PIK-III (2.5, 5 and 10 μM). 24 hours later, cells were lysed to conduct Western blot to measure FN (FN1), Col1 (COL1A1/COL1A2) and αSMA (ACTA2) expression (n = 3). **B)** Cells were mixed with collagen 1 solution as described in Methods and treated with TGF-β1 (10 ng/ml) in the presence or absence of PIK-III (5 μM). Gel size was measured at 0 and 24 h after collagen gelation (n = 3 for each condition). **C)** Cells were treated as described in (A). After treatment, cells were lysed for total RNA isolation. mRNA levels of collagen1a1 (*COL1A1*), fibronectin1 (*FN1*) and αSMA (*ACTA2*) were measured by qRT-PCR (n = 3 for each condition). Data are mean ± SEM. P < 0.05; significant comparisons by one-way ANOVA:* vs. unstimulated, † vs. TGF-β1 alone.

HDFs were stimulated with TGF-β1 (10 ng/ml) for 24 hours in the presence or absence of PIK-III (2.5, 5, and 10 μM). As expected, TGF-β1 stimulation induced cell contraction and led to a significant increase in Col1, FN and αSMA expression at both protein and mRNA levels (Fig 1A). In contrast, 5 μM of PIK-III significantly inhibited profibrotic responses in TGF-β1-induced HDFs (Fig 1A–1C) without affecting cell viability (S1 Fig in S1 File).

### PIK-III inhibits TGF-β1-induced p38 activation in HDFs

**Fig 2:**
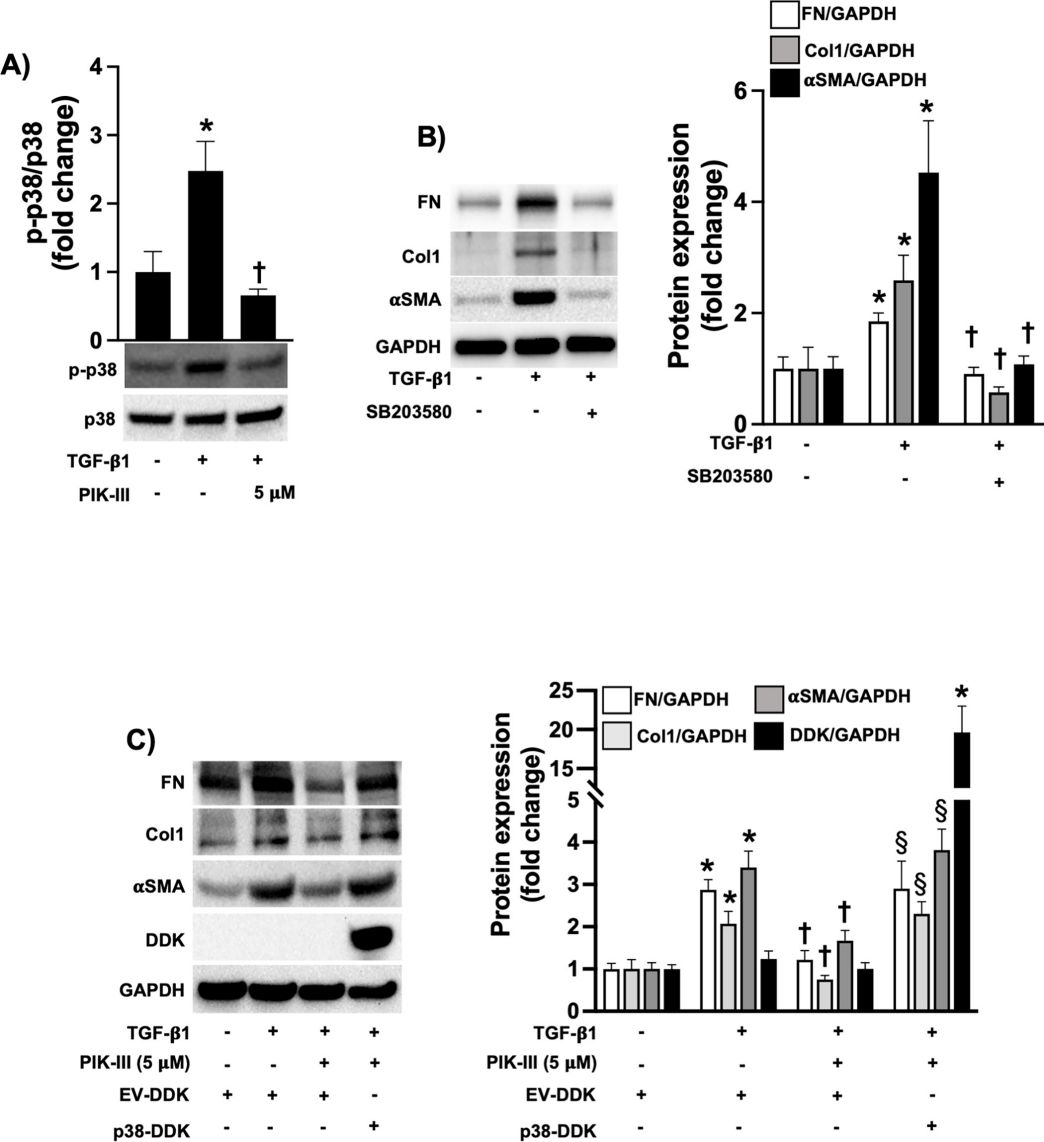
PIK-III attenuates the phosphorylation of p38 in activated HDFs. **A)** HDFs were stimulated with TGF-β1 (10 ng/ml) in the presence or absence of PIK-III (5 μM). 24 hours later, cells were lysed for Western blot to measure phospho-p38 (Thr180/Tyr182) (n = 3). **B)** HDFs were stimulated with TGF-β1 (10 ng/ml) in the presence or absence of SB203580 (10 μM). 24 hours later, cells were lysed for Western blot to measure Fibronectin, Type I Collagenand αSMA expression (n = 3). **E)** HDFs were lentivirally transfected with pLenti-C-Myc-DDK (empty vector, EV) or pLenti-p38-Myc-DDK (p38-DDK). Then, cells were stimulated with TGF-β1 (10 ng/ml) in the presence or absence of PIK-III (5 μM). 24 hours later, cells were lysed for Western blot. Data are mean ± SEM. P < 0.05; significant comparisons by one-way ANOVA:* vs. unstimulated, † vs. TGF-β1 alone.

Since PIK-III is considered a selective inhibitor of PIK3C3, we subsequently assessed if the observed anti-fibrotic effects were a result of PIK-III-induced inhibition of PIK3C3. To test this, we treated HDFs with the PIK3C3 inhibitors SAR405 and autophinib expecting that they will mimic PIK-III-mediated effect. However, to our surprise, neither SAR405 nor autophinib significantly attenuated profibrotic responses in TGF-β1-induced HDFs (S2A Fig in S1 File). Likewise, genetic depletion of PIK3C3 by lentiviral transfection of shRNA (S2B Fig in S1 File) did not regulate ECM and αSMA protein expression in the presence or absence of TGF-β1 (S2C Fig in S1 File), suggesting that PIK-III exerted its anti-fibrotic effect in fibroblasts through another signaling pathway. Thus, we examined if PIK-III can regulate MAPK activation in dermal fibroblasts. To test this, we used MAPK phosphorylation antibody array, which can detect the activation of 17 proteins (S4 Fig in S1 File). Our results showed that TGF-β1 treatment stimulated the activation (phosphorylation) of different signaling proteins, including glycogen synthase kinase 3 (GSK3), c-Jun N-terminal Kinases (JNK), p38 and p53 (S4 Fig in S1 File). p38 was one of the protein whose phosphorylation was reduced by PIK-III treatment in TGF-β1-induced HDFs using antibody array assay (S4 Fig in S1 File). Western blot confirmed PIK-III inhibition of p38 activation (Fig 2A). To validate that PIK-III inhibitory effect on p38 activation attributes to its anti-fibrotic effects, we then treated HDFs with the p38 inhibitor (SB203580). HDF exposure to SB203580 mimicked our findings upon PIK-III treatment (Fig 2B). Finally, we found that p38 overexpression reversed inhibitory effect of PIK-III on αSMA and ECM expression suggesting that p38 signaling plays an important role in the anti-fibrotic effects exerted by PIK-III treatment (Fig 2C).

### PIK-III attenuates bleomycin-induced dermal fibrosis in mice

**Fig 3:**
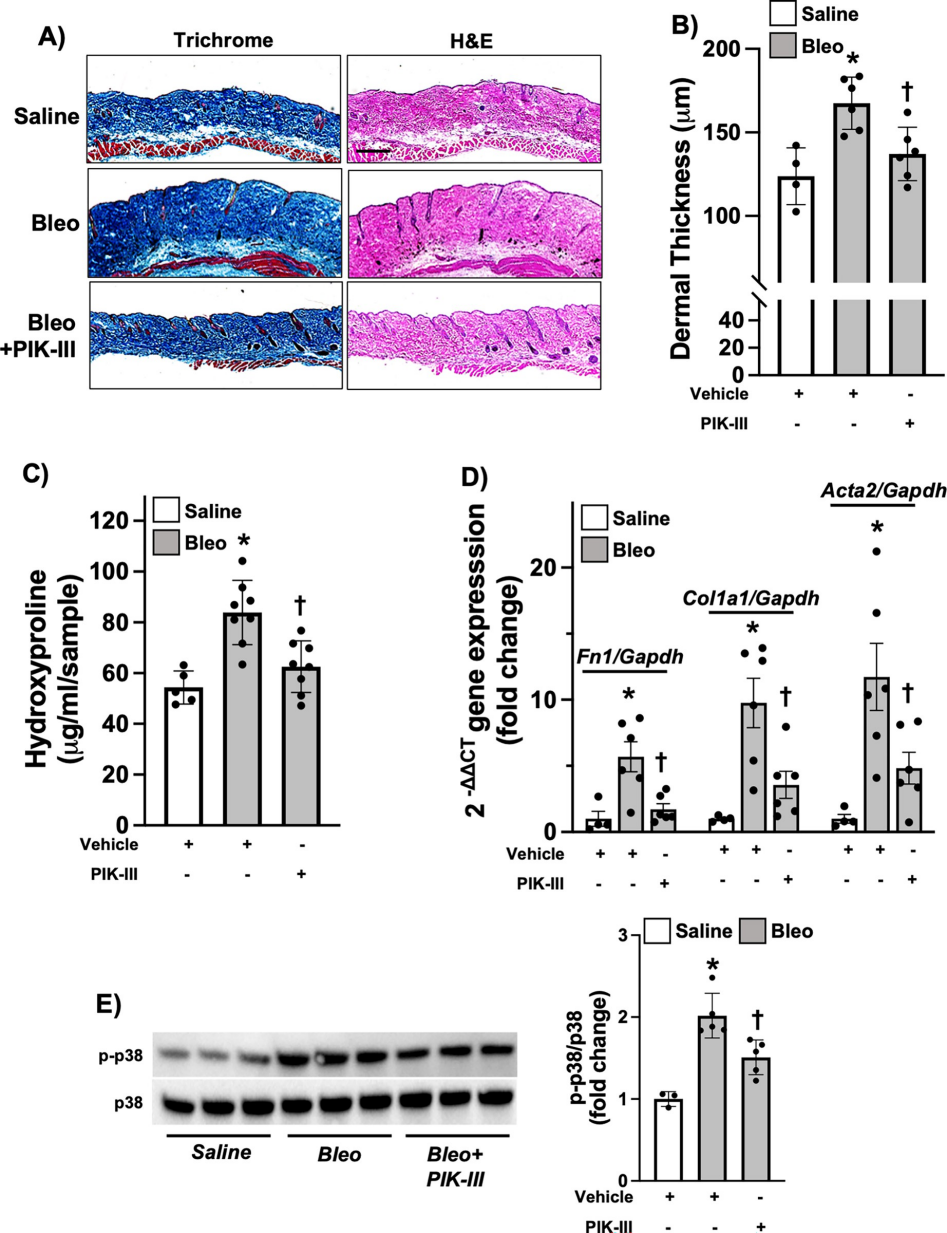
PIK-III inhibits dermal fibrosis in mice. Mice were subjected to Bleo-induced dermal fibrosis with or without treatment with PIK-III (5 mg/kg) as described in Methods. **A and B)** 28 days after first Bleo administration, 6 mm skin biopsies were harvested, fixed and subjected to Trichrome and H&E staining. Dermal thickness was measured in H&E stained skin samples using ImageJ software (n = 4 or 6). **C)** Hydroxyproline content was measured in the 6 mm punch skin biopsies of Vehicle (*n* = 8) and PIK-III treated mice (*n* = 8) exposed to Bleo (gray bars) and Vehicle treated mice exposed to PBS (*n* = 5) (white bar). **D)** Gene expression of *Fn1* and *Col1a1*, and *Acta2* in lesional skin samples was measured using qRT-PCR (*n* = 4 or 6). **E)** Skin samples were homogenized and lysed for western blot to measure phospho-p38 and total p38 (n = 3 or 5). Data are mean ± SEM. P < 0.05; significant comparisons by one-way ANOVA:* vs. Vehicle/PBS, † vs. Bleo alone.

We next examined whether PIK-III regulates dermal fibrosis *in vivo*. Subcutaneous administration of bleomycin (Bleo) into mice is a widely accepted experimental model of dermal fibrosis and scleroderma [17]. As expected, administration of Bleo led to significant dermal thickening and collagen deposition, measured by hydroxyproline assay and trichrome staining, in mice (Fig 3A–3C). Consistent with our *in vitro* results (Fig 1), exposure to 5 mg/kg of PIK-III in mice significantly ameliorated dermal fibrosis by downregulating profibrotic gene expression induced by Bleo (Fig 3A–3D). We also observed significant inhibition of p38 activation in PIK-III treated samples compared to Bleo alone.

### PIK-III inhibits lung fibroblast activation and regulates pulmonary fibrosis in mice

**Fig 4:**
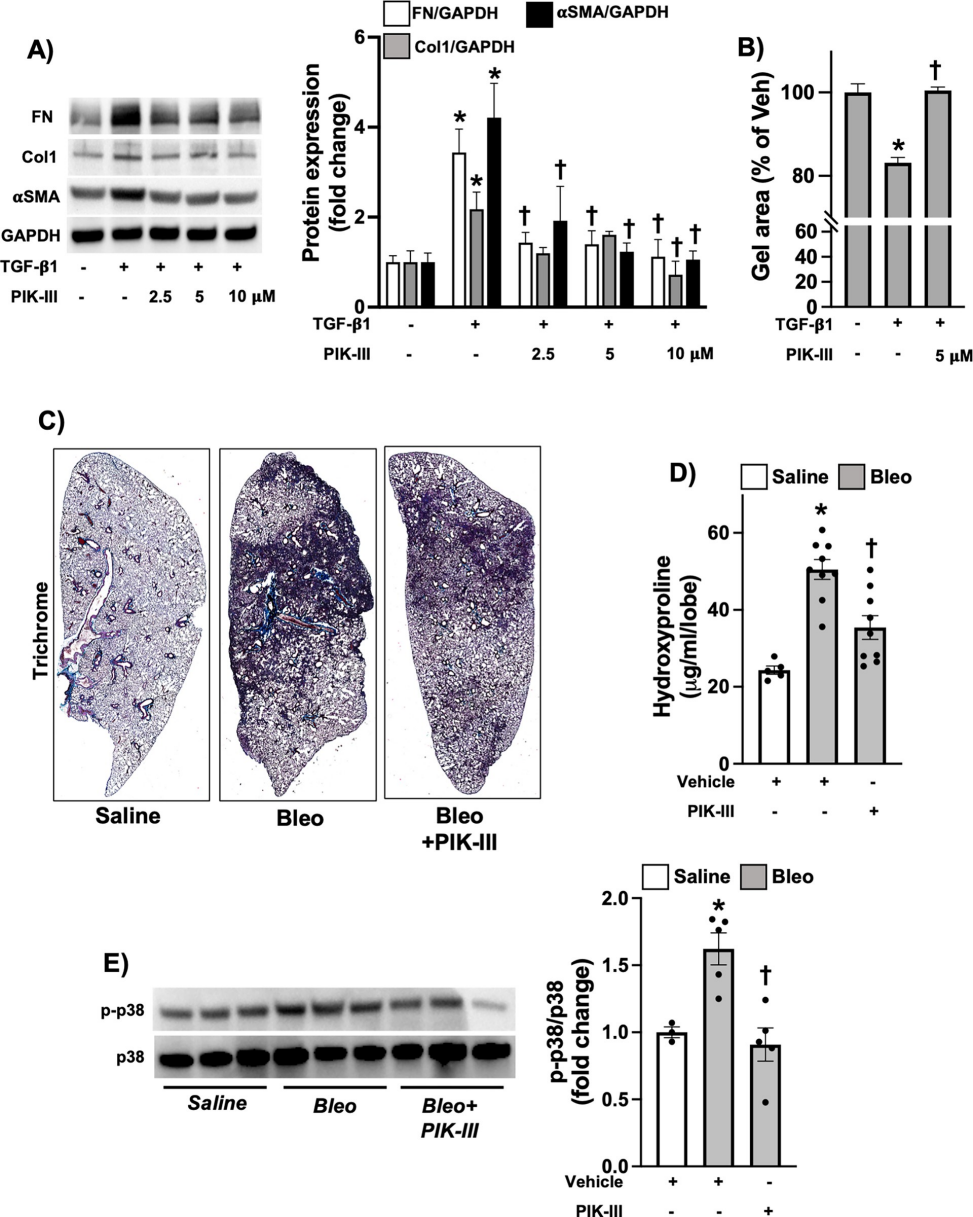
PIK-III inhibits lung fibroblast activation and pulmonary fibrosis. **A)** HLFs were stimulated with TGF-β1 (10 ng/ml) in the presence or absence of PIK-III (2.5, 5 and 10 μM). 24 hours later, cells were lysed for Western blot to measure fibronectin (FN1), Collagen 1 (COL1A1/COL1A2) and αSMA (ACTA2) expression (n = 3). **B)** HLFs were mixed with collagen 1 solution as described in Methods and treated with TGF-β1 (10 ng/ml) in the presence or absence of PIK-III (5 μM). Gel size was measured at 0 and 24 h after collagen gelation (n = 3). **C and D)** Mice were subjected to Bleo-induced lung fibrosis with or without treatment with PIK-III (5 mg/kg) as described in Methods. 21 days after Bleo administration, lungs were harvested and subjected to Trichrome stain (C) and hydroxyproline assay (D) (*n* = 5–9). **E)** Lung samples were homogenized and lysed for western blot to measure phospho-p38 and total p38 (n = 3 or 5). Data are mean ± SEM. P < 0.05; significant comparisons by one-way ANOVA:* vs. unstimulated or Vehicle/PBS, † vs. TGF-β1 or Bleo alone.

Finally, we examined whether PIK-III treatment provides similar anti-fibrotic effects in pulmonary fibrosis settings. Our results showed that TGF-β1-induced human lung fibroblasts (HLFs) produced significantly lower levels of fibronectin, collagen and αSMA upon PIK-III exposure (Fig 4A and 4B). Similarly, PIK-III therapy after intratracheal administration of Bleo, a well-known experimental model of lung fibrosis, also attenuated lung fibrosis and p38 activation at day 21 (Fig 4C–4E, S5 Fig in S1 File). Collectively, our findings suggest that PIK-III exerts potent anti-fibrotic effects in both dermal and lung experimental models of fibrosis.

## Discussion

PIK3C3 is believed to be the only class III PI-3-kinase in mammals responsible for phosphorylating phosphatidylinositol at the 3′ position on the inositol ring and producing phosphatidylinositol-3-phosphate (PI(3)P) [18]. PIK3C3 plays critical roles in a wide range of cellular processes, including autophagy and lysosome trafficking, thus serving as potential target for therapeutic intervention in disorders where these processes are dysregulated [18–20]. Several PIK3C3 inhibitors have been developed which demonstrate potent therapeutic effect in different experimental models of various diseases [12, 13, 15, 16, 20–22]. To this end, we attempted to determine the role of PIK3C3 in dermal fibrosis using genetic and pharmacological approaches to PIK3C3 loss of function. To our surprise, we did not find significant differences in fibroblast activation in PIK3C3 silenced dermal fibroblasts compared to scramble transfected cells, suggesting that fibroblasts do not require PIK3C3 to mediate profibrotic gene expression (S2C Fig in S1 File). It is worth noting that a profibrotic phenotype of fibroblasts is not limited by overexpression of ECM proteins and myofibroblast differentiation [23]. It is known that fibroblasts isolated from fibrotic tissue also have increased migratory and invasive properties as well as resistance to pro-apoptotic stimuli, such as Fas ligand (FasL) [24, 25]. Thus, the potential role of PIK3C3 in fibroblast migration, invasion and resistance to apoptosis should be further investigated. However, a pharmacological inhibitor of PIK3C3, PIK-III, but not SAR405 or autophinib, demonstrated anti-fibrotic effects in dermal and lung fibroblasts as well as in animal models of skin and lung fibrosis (Figs 1, 3 and 4). As, one of the major downstream activities of PIK3C3 is the induction of autophagy, we observed that PIK-III, SAR405 and autophinib potently reduced autophagy in PIK3C3 overexpressing cells (S3 Fig in S1 File) [13, 15, 20, 21, 26]. These findings suggest that all three inhibitors downregulate PIK3C3 activity but only PIK-III may downregulate fibroblast activation in profibrotic settings. Furthermore, PIK3C3 deficiency did not regulate fibroblast’s ECM production and myofibroblast transformation, thus we hypothesized that PIK-III may regulate other signaling pathways to exert its anti-fibrotic effects. Using a phosphorylation antibody array, we found that PIK-III could inhibit p38 activation, which was also confirmed by western blot (Fig 2A). Furthermore, SB203580 (a specific p38 inhibitor) attenuated dermal fibroblast activation in response to TGF-β1, in a similar manner to PIK-III (Fig 2B). Finally, p38 overexpression could significantly ameliorate PIK-III-mediated antifibrotic effect in HDFs (Fig 2C). These results suggest that PIK-III exerts its beneficial effects via regulation of p38 activation.

Indeed, previous reports have established that p38 plays a key role in TGF-β1-mediated profibrotic gene expression by regulating the activation of different transcription factors in fibroblasts, such as activating transcription factor-2 (ATF-2), Sp1/2 and activator protein-1 (AP-1) [27–30]. In preclinical animal models, inhibition of p38 demonstrated potent therapeutic effects against kidney, liver, pulmonary and skin fibrosis [31–34]. Finally, p38α deficiency in fibroblasts blocked the transition of fibroblasts into myofibroblasts and attenuated cardiac fibrosis in mice [35]. Whereas the overexpression of mitogen-activated protein kinase kinase 6 (MKK6), an upstream kinase of p38 signaling, in fibroblasts has led to spontaneous development of fibrotic remodeling in the heart, lung and kidneys [35]. Taken together, previous reports and our findings strongly suggest the potential therapeutic benefit of p38 inhibition in different models of organ fibrosis.

Overall, we found that PIK-III possesses potent anti-fibrotic effects in experimental models of skin and lung fibrosis by regulating the fibroblast profibrotic responses. Thus, PIK-III and its derivatives, *e*.*g*. compound 19, can be considered as potential candidates to treat scleroderma and scleroderma-associated ILD [21, 36].

## Materials and methods

### Reagents

PIK-III, SAR405 and SB203580 were obtained from Cayman Chemicals (Ann Arbor, MI). Autophinib was acquired from Sigma-Aldrich. Recombinant TGF-β1 was obtained from Sino Biological (Cat. # 10804-HNAC). All other reagents were acquired from Sigma-Aldrich.

### Cells

Human Dermal Fibroblasts (HDFs) from healthy donors were kindly provided by Dr. Assassi (UTHealth Medical Center, Houston, TX) through Material Transfer Agreement (MTA) and used under Institutional Review Board (IRB)-approved protocol (Protocol number: H-50814) at Baylor College of Medicine (BCM) on May 2022. Human Lung Fibroblasts from control subjects were obtained by IRB-approved protocol (Protocol number: H-46823) at BCM between November 2021-December 2022. All human cells were deidentified prior to transfer to Dr. Tsoyi’s laboratory. Fibroblasts were cultured in complete media (DMEM; Corning) containing 10% FBS (Corning), 100 IU of penicillin and 100 μg/ml streptomycin (Corning), and 292 μg/ml L-glutamine (Corning) in humidified incubators at 37°C and 10% CO_2_. Cells were periodically tested for mycoplasma contamination using a commercially available kit (ThermoFisher Scientific).

### Quantitative real time-polymerase chain reaction (qRT-PCR)

Total RNA was isolated using the RNeasy Mini Kit (Qiagen) according to the manufacturer’s protocol. Quantitative RT-PCR with SYBR Green Master Mix (Bio-Rad Laboratories, Hercules, CA, USA) was performed by use of the StepOnePlus Real-Time PCR System (Applied Biosystems). The relative quantity of target mRNA was calculated by use of the CT method, or 2^–ΔΔCT^, as previously described [37] and normalized by use of glyceraldehyde 3-phosphate dehydrogenase (GAPDH) as an endogenous control. Primer’s sequences are described in S6 Table in S1 File.

### Western blot

Polyacrylamide gel electrophoresis and immunoblotting were performed according to standard methods as previously described [38]. Quantification of immunoreactive protein bands was performed with the computer software ImageJ and was expressed as a ratio of fold change band intensity with respect to the loading control. Antibodies to αSMA, Col1 and FN were obtained from Abcam (Cambridge, MA); GAPDH from Santa Cruz Biotechnology (Santa Cruz, CA); phospho-p39 (Thr180/Tyr182) and total p38 from Cell Signaling Technology (Berkeley, CA). Full list of antibodies and their dilutions are described in S6 Table in S1 File.

### Cell contraction assay

Cell contraction of human dermal or lung fibroblasts was performed as previously described [39].

### Animal model of Bleo-induced dermal fibrosis

Eight to ten weeks old C57BL/6J mice were subcutaneously (SC) injected with 50 μl of 1 mg/ml of bleomycin sulfate (Bleo) from Cayman Chemicals diluted in sterile 1xPBS on alternate days for 28 days. 12 days after first Bleo injection, mice were randomized and intraperitoneally (IP) treated with PIK-III (5 mg/kg) or vehicle (DMSO) every other day until the end of experiment (8 injections in total). At day 28, 6 mm biopsies were taken at the site of Bleo administration.

### Animal model of Bleo-induced lung fibrosis

Eight to ten weeks old C57BL/6J mice were intratracheally (IT) injected with Bleo (0.3 mg/kg). 10 days after Bleo administration, mice were randomized and treated with PIK-III (5 mg/kg) or vehicle (DMSO) every day for 5 days. 21 days later, lungs were harvested, and fibrosis was assessed.

### Hydroxyproline assay

Collagen deposition was measured by hydroxyproline assay and quantified as previously described [40, 41]. Each sample was assayed in triplicate. Data are reported as micrograms of hydroxyproline from upper right lobe of the lung or 6 mm skin biopsy.

### Histology

Skin and lung sections were fixed by inflation with buffered 10% formalin solution and embedded in paraffin. Thin (4 μm) sections were deparaffinized and rehydrated. Tissue slides were then stained by hematoxylin-eosin (H&E) and Masson’s Trichrome staining to evaluate histopathologic changes and collagen deposition, respectively [41].

### Statistical analysis

Data are expressed as mean ± standard error the mean. For comparisons between two groups, we used Student’s unpaired t test. One-way analysis of variance followed by Newman-Keuls or Tukey’s post-test analysis was used for analysis of more than two groups. The numbers of samples per group (n), or the numbers of experiments, are specified in the figure legends. Statistical significance was defined as p<0.05.

## Supporting information

S1 File Supplemental methods, supplemental figures and raw images. (DOCX)
