# Supplementary material for: PIK-III exerts anti-fibrotic effects in activated fibroblasts by regulating p38 activation
Source: PLoS One. 2024 Sep 6;19(9):e0306624. doi: 10.1371/journal.pone.0306624 (PMC11379285; doi:10.1371/journal.pone.0306624)
Supplement: S1 File — (DOCX) [file pone.0306624.s001.docx]

**SUPPLEMENTAL INFORMATION**

**SUPPLEMENTAL METHODS**

**3-(4,5-dimethylthiazol-2-yl)-2,5-diphenyl-2H-tetrazolium bromide (MTT) assay**

Cell viability was determined using the MTT reagent. The cells were seeded at 1 × 10^4^ cells/well in 24-well plates. After different treatments, MTT solution was added to each well (0.1 mg/ml) and incubated for 4 h. The supernatant was aspirated, and the formazan crystals in each well were dissolved in 200 μl dimethyl sulfoxide (DMSO) for 30 min at 37 °C, and the plates were read at 570 nm.

**Lentiviral transfection**

PLKO.1 plasmids carrying either the human PIK3C3/VPS34 shRNA target sequence

GAGGCAAATATCCAGTTATAT (consortium number TRCN0000196290) or a scramble (Scr) sequence were purchased from Sigma-Aldrich (St. Louis, MO). Lentiviral particles were generated by use of a commercially available packaging mix (Cat. #: SHP001; Sigma-Aldrich, St Louis, MO) in human embryonic kidney 293 T (HEK293T) cells according to the manufacturer's instructions. Cells were infected with the lentiviral particles and stably selected by use of puromycin (10 μg/mL). pLenti-C-Myc-DDK (empty vector, EV, Cat. #: PS100064V) or pLenti-p38-Myc-DDK (p38-DDK, Cat. #: RC206605L1V) lentiviral particles were purchased from Origene Technologies, Inc. and cells were transfected as described in the manual. Lenti-CMV-Blank-CBH-GFP-2A-Puro (empty vector, EV-GFP, Cat. #: LVP690) and Lenti-CMV-PIK3C3-CBH-GFP-2A-Puro (PIK3C3-GFP, Cat. #: LV7-36679061) lentiviral particles were purchased from Applied Biological Materials (ABMGood) and cells were transfected as described in manual. Transfection efficiency was confirmed by measuring GFP-positive cells using flow cytometry.

**Phosphorylation antibody array**

Phosphorylation antibody array kit was obtained from Abcam (Cat. # ab21106) was performed according to manufacture’s guidelines.

**SUPPLEMENTAL FIGURE LEGENDS**

**S1 Figure. Effect of PIK-III on cell viability.** HDFs were seeded into 24 well plate as described in Supplemental Methods. Then, cells were treated with PIK-III (2.5, 5, 10 and 20 μM) (n=4). 24 hours later, cells were subjected to cell viability MTT assay as described in Supplemental Methods. Data are mean ± SEM. P < 0.05; significant comparisons by one-way ANOVA:* vs. untreated.

**S2 Figure. Effect of VPS34 on profibrotic responses in activated HDFs. A)** HDFs were stimulated with TGF-β1 (10 ng/ml) in the presence or absence of PIK-III (5 μM), SAR405 (5 μM), and autophinib (5 μM). 24 hours later cells were lysed to Western blot to measure FN, Col1 and αSMA expression (n=3).  **B)** HDFs were lentivirally transfected with Scr or shVPS34 and selected with puromycin (10 μg/mL) as described in Supplemental Methods. After selection, cells were lysed and mRNA levels of VPS34 was measured by qRT-PCR as described in Methods. **C)** Scr and shVPS34 stably transfected HDFs were stimulated with TGF-β1 (10 ng/ml). 24 hours later cells were lysed to Western blot to measure FN, Col1 and αSMA expression (n=3).

**S3 Figure. PIK-III, SAR405 and Autophinib reduce PIK3C3-induced autophagy.** HEK293T cells were lentivirally transfected with Lenti-CMV-Blank-CBH-GFP-2A-Puro (empty vector, EV-GFP) or Lenti-CMV-PIK3C3-CBH-GFP-2A-Puro (PIK3C3-GFP). Then cells were treated with PIK-III (5 μM), SAR405 (5 μM), and autophinib (5 μM) for 24 hours. Transfection efficiency was measured by quantifying GFP positive cells using flow cytometry (n=3) **(A)** LC3A/B-I and -II isoforms and PIK3C3 expression were measured by Western blot (n=3) **(B)**.

**S4 Figure. Effect of PIK-III phosphorylation of different signaling proteins in activated HDFs.** HDFs were stimulated with TGF-β1 (10 ng/ml) in the presence or absence of PIK-III (5 μM). 24 hours later cells were harvested and 200 μg of protein lysate was used for each condition. Phosphorylation antibody array was performed as described in Supplemental Methods. The experiment was repeated twice.

**S5 Figure. H&E staining of mouse lungs.** Bleo-induced lung fibrosis and PIK-III treatment was performed as described in Figure 4 C. H&E staining of the lungs were performed as described in Methods.

**
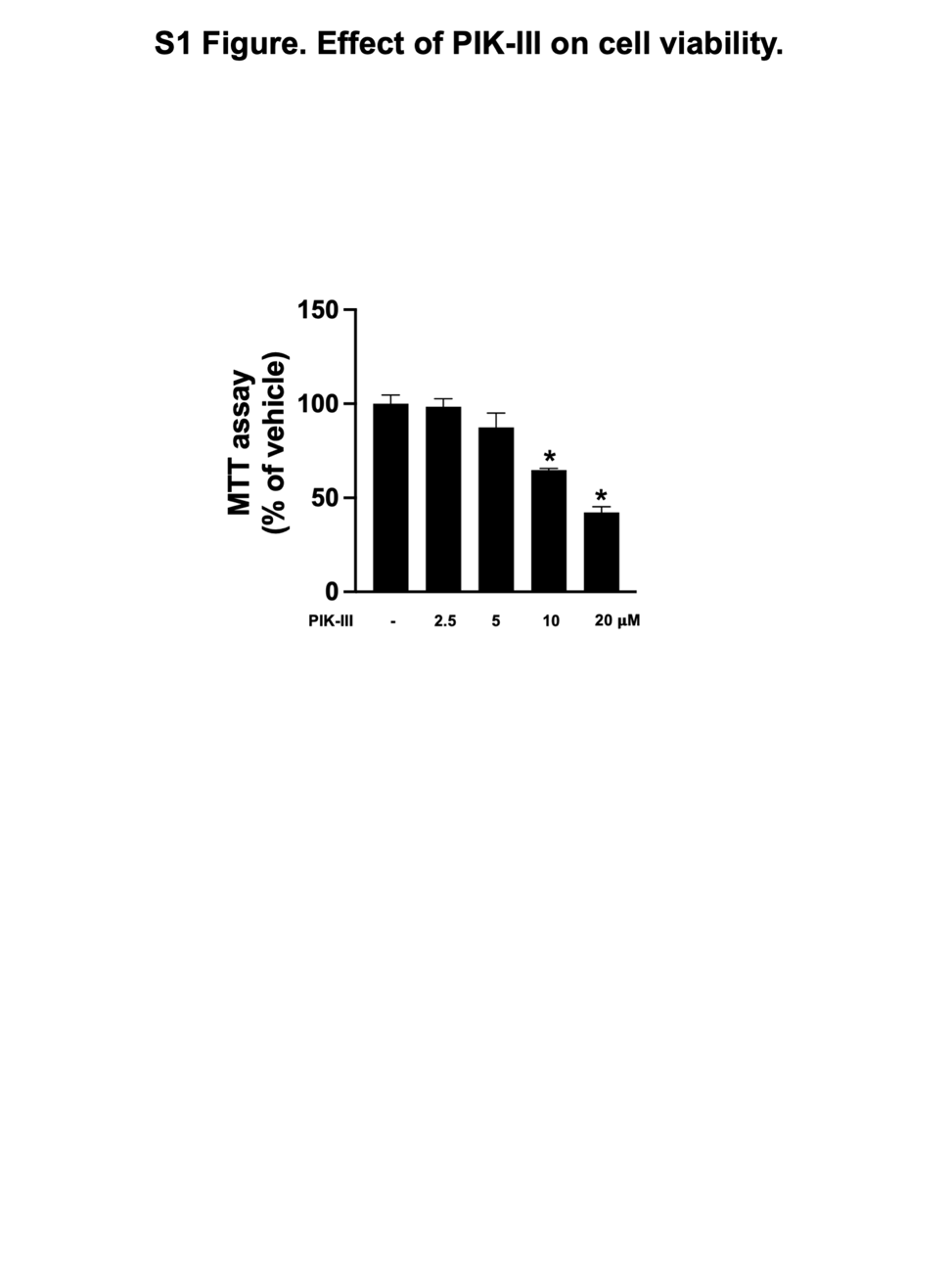
**

**
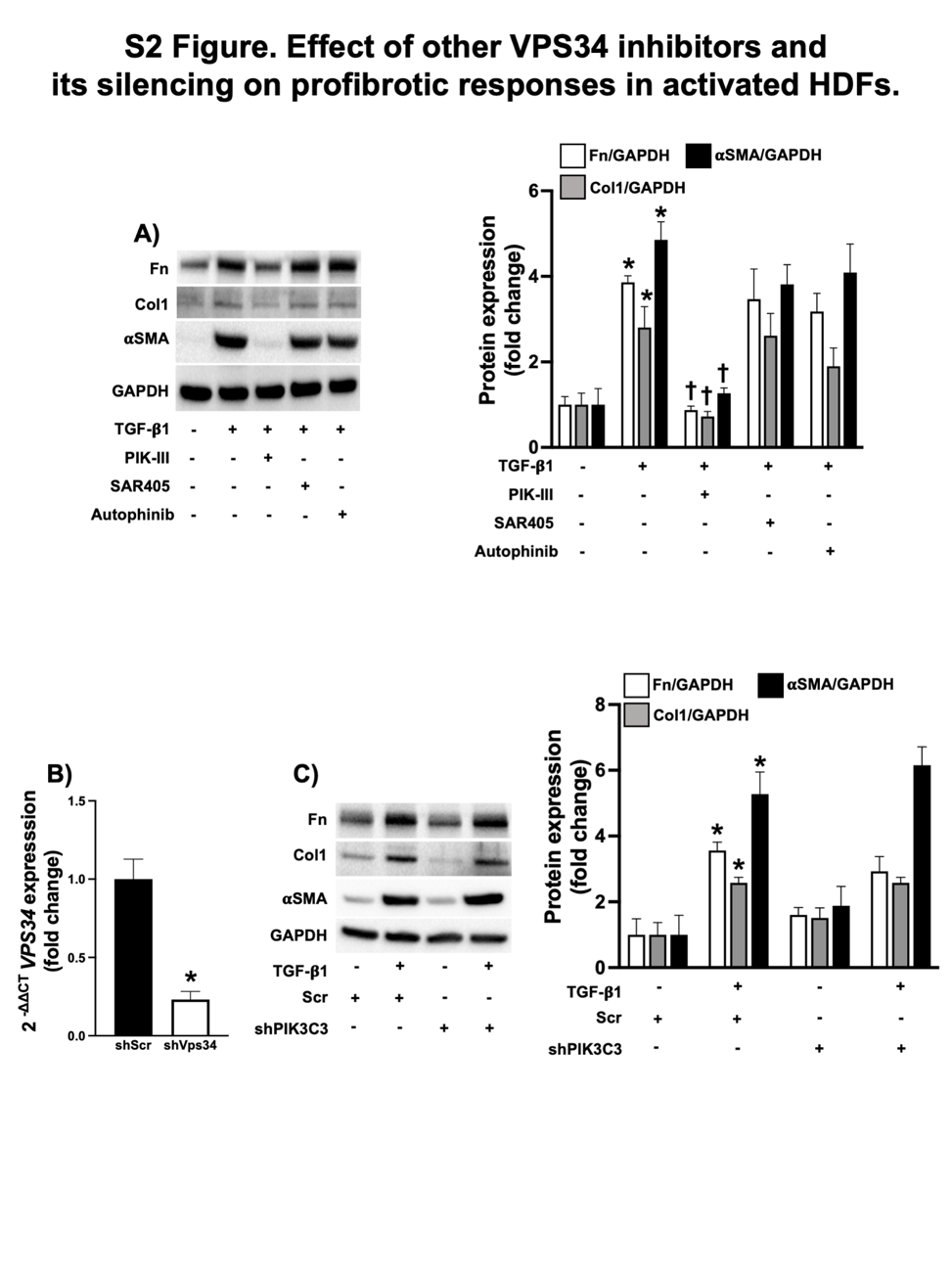
**

**
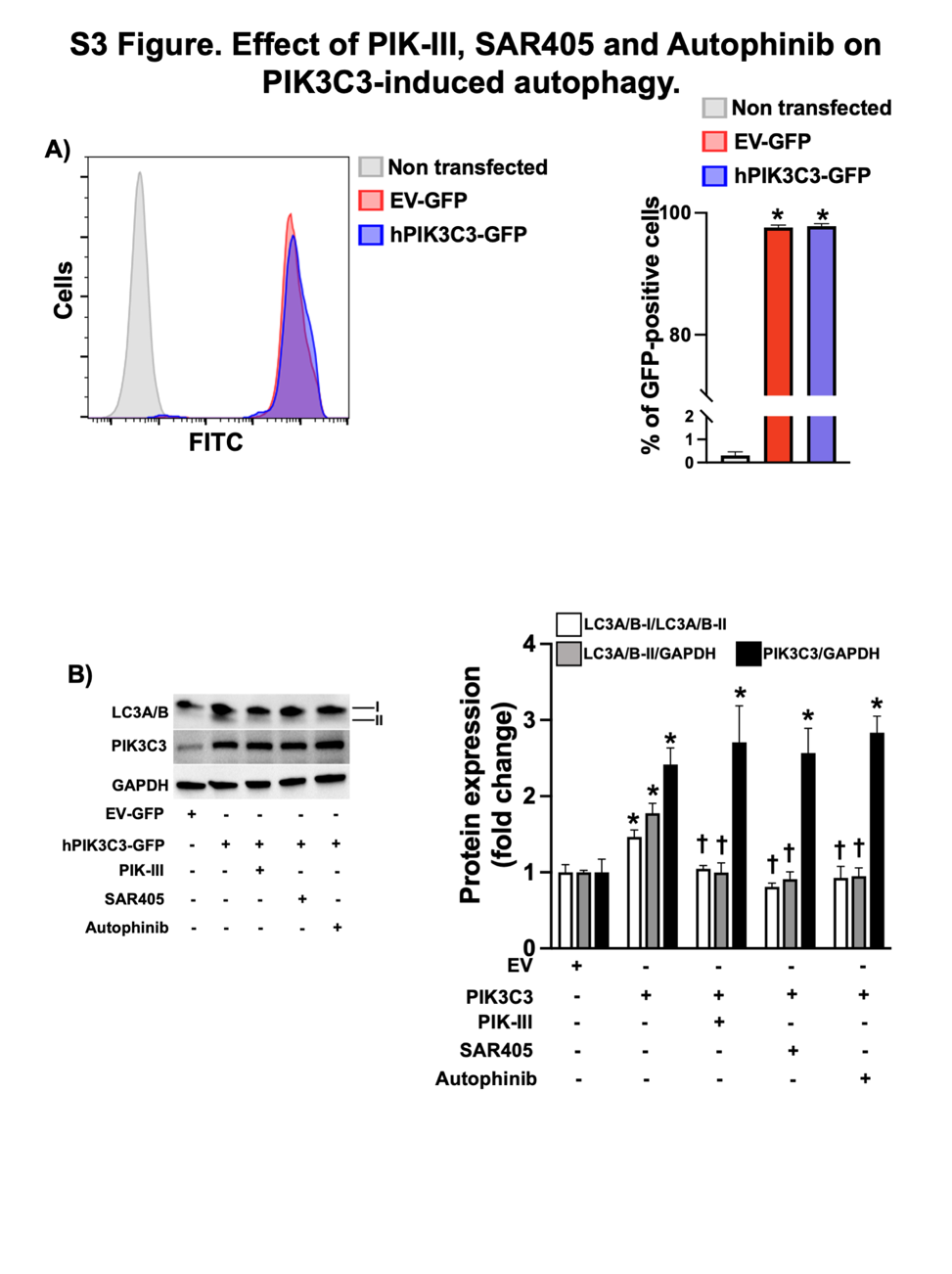
**

**
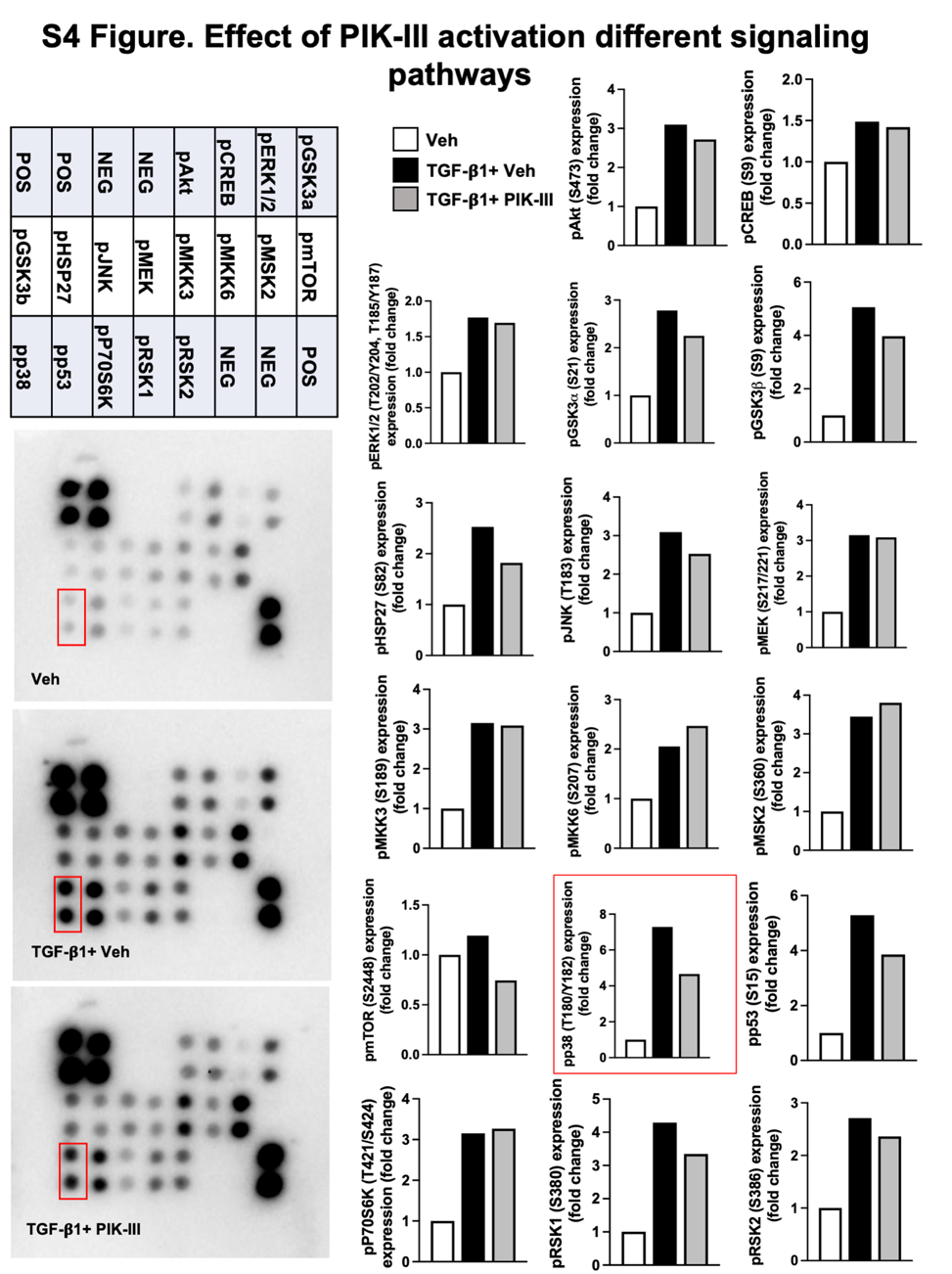
**

**
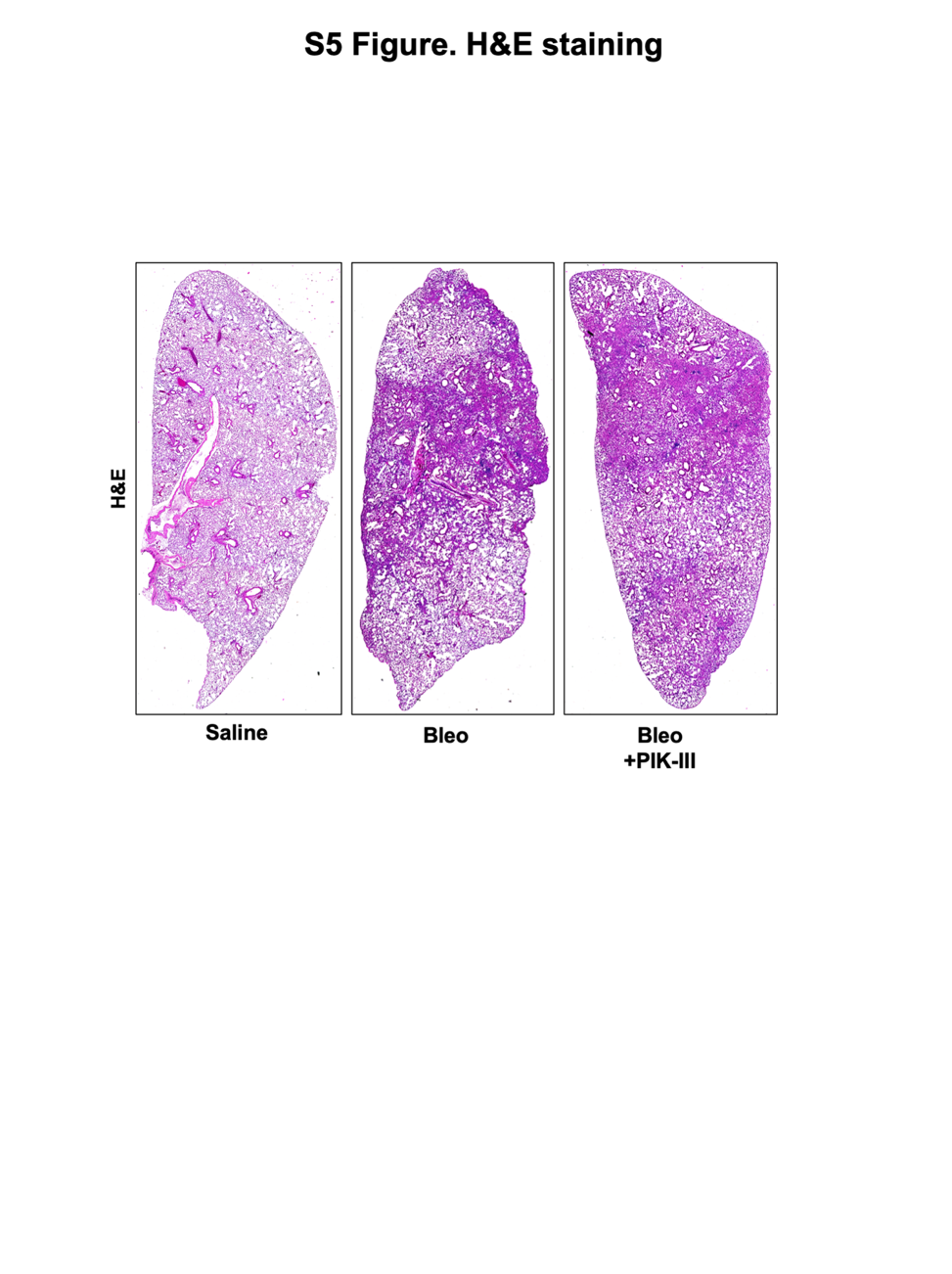
**

**
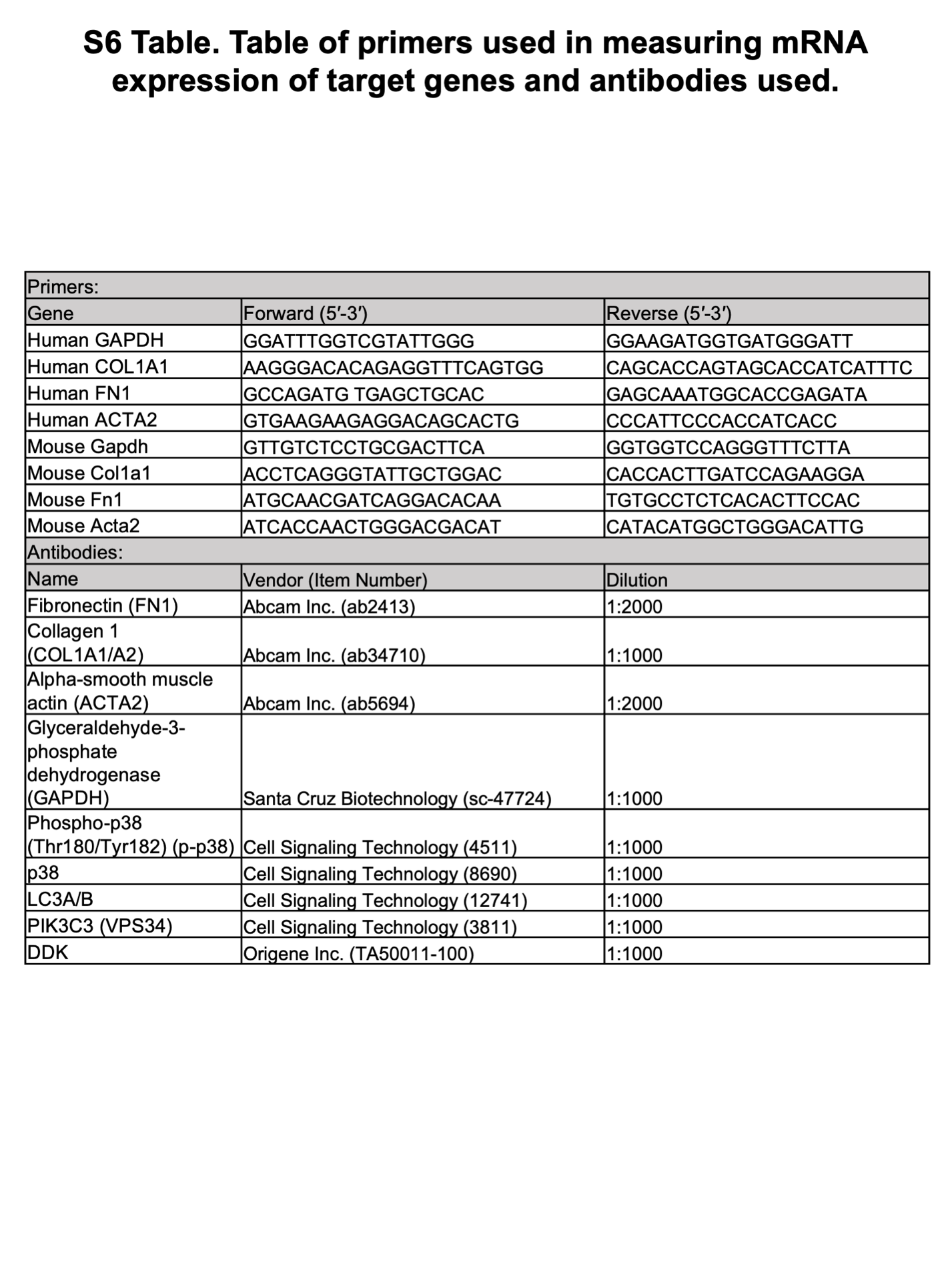
**

**
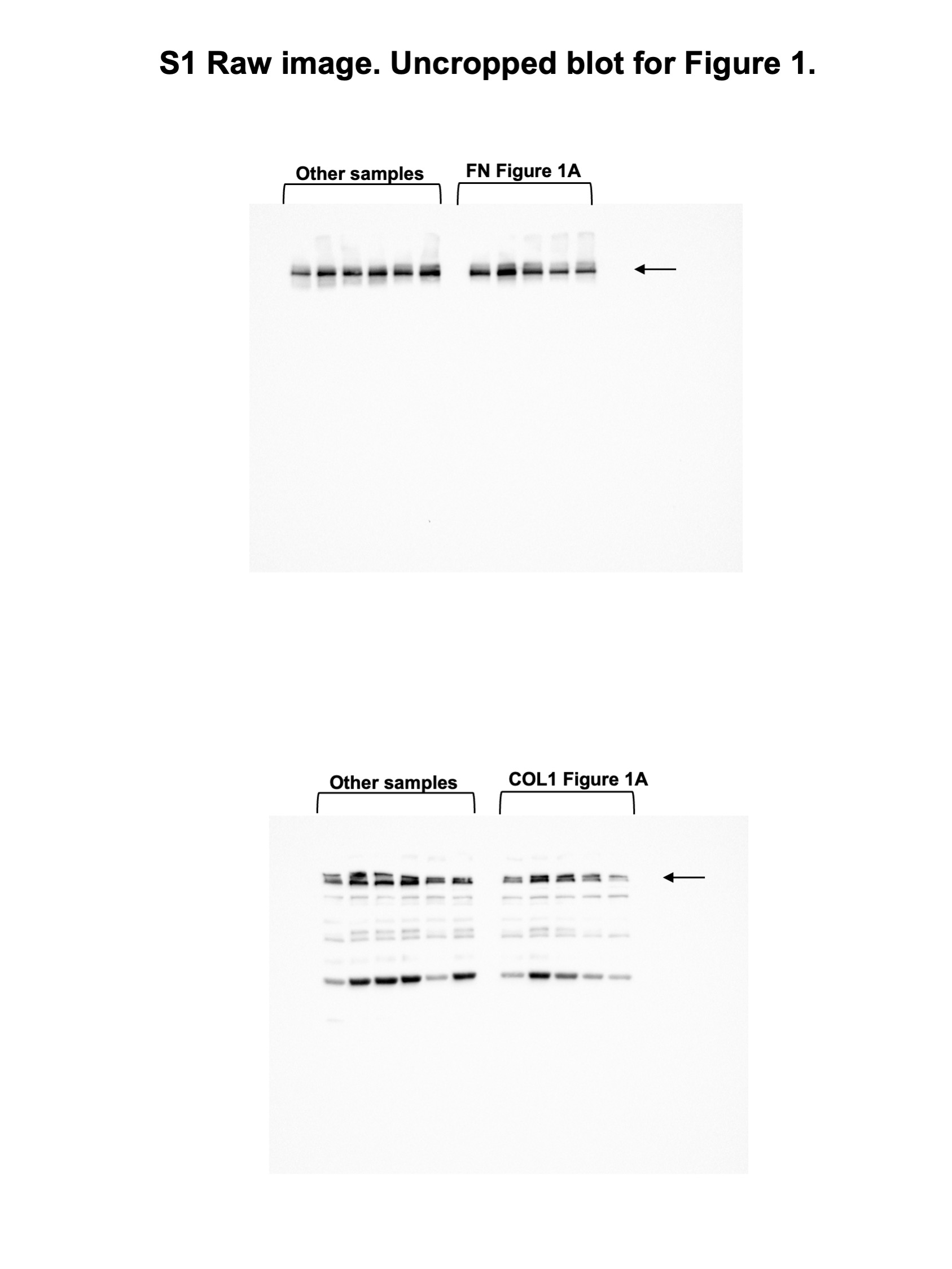
**

**
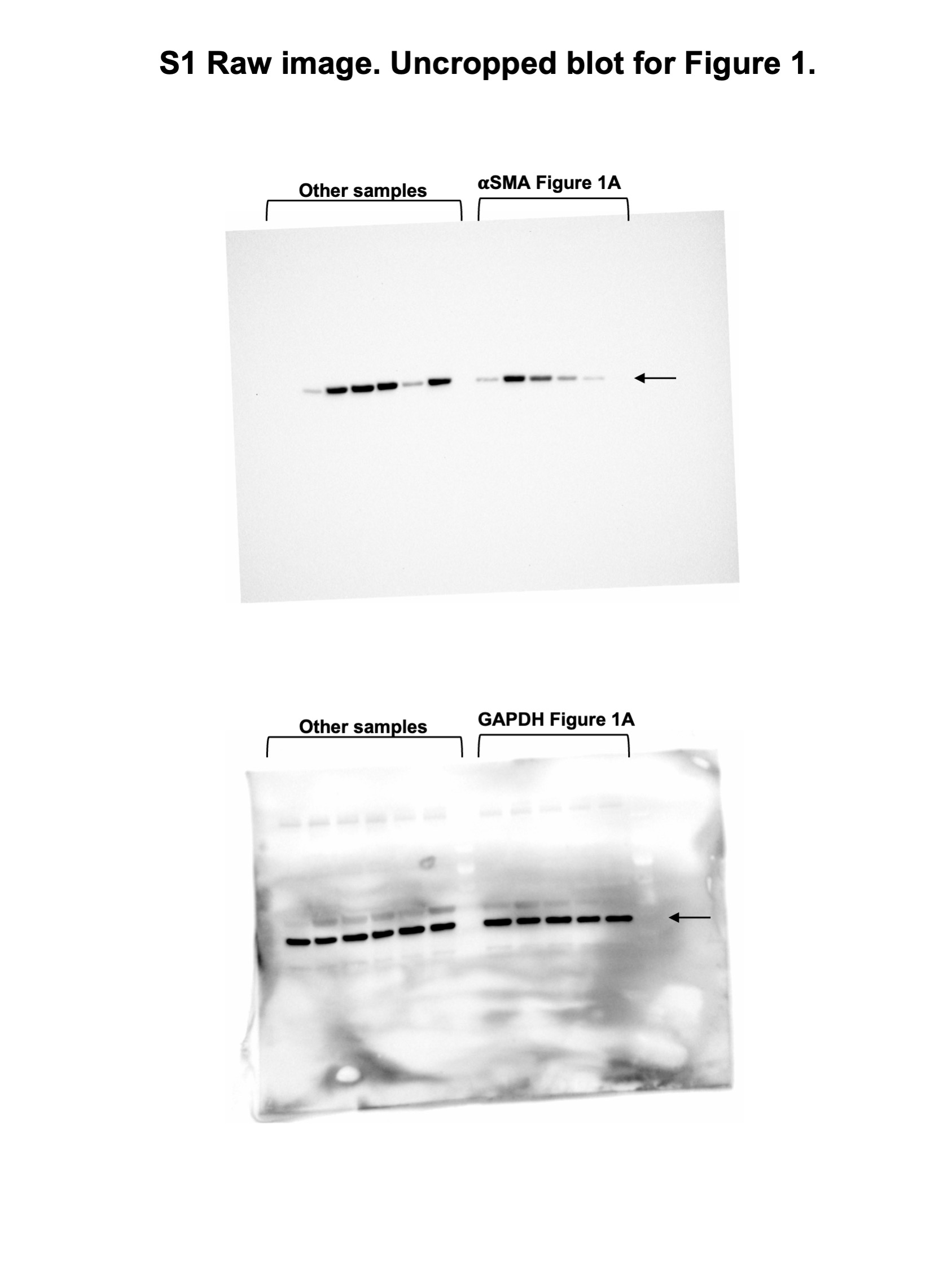
**

**
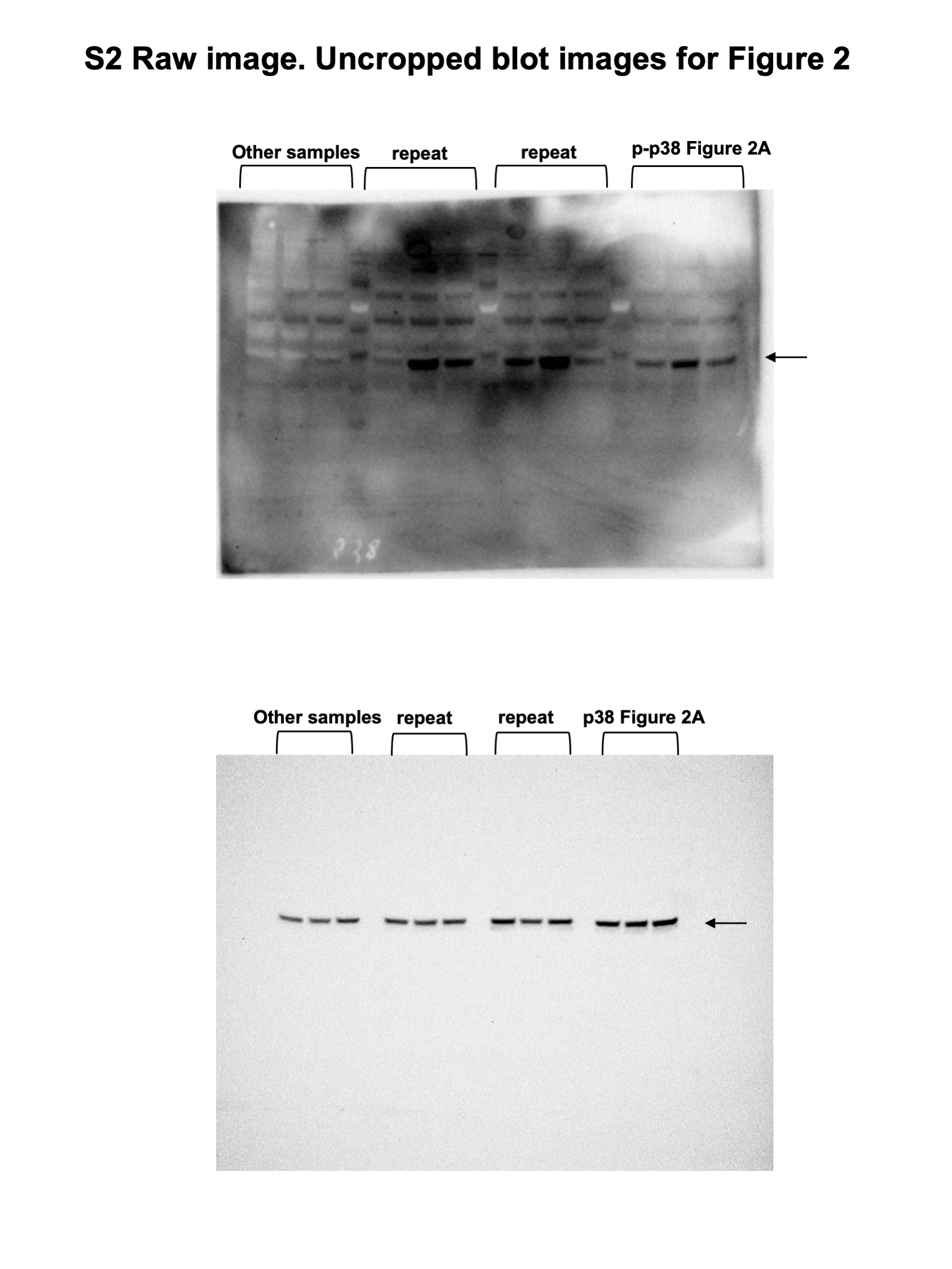
**

**
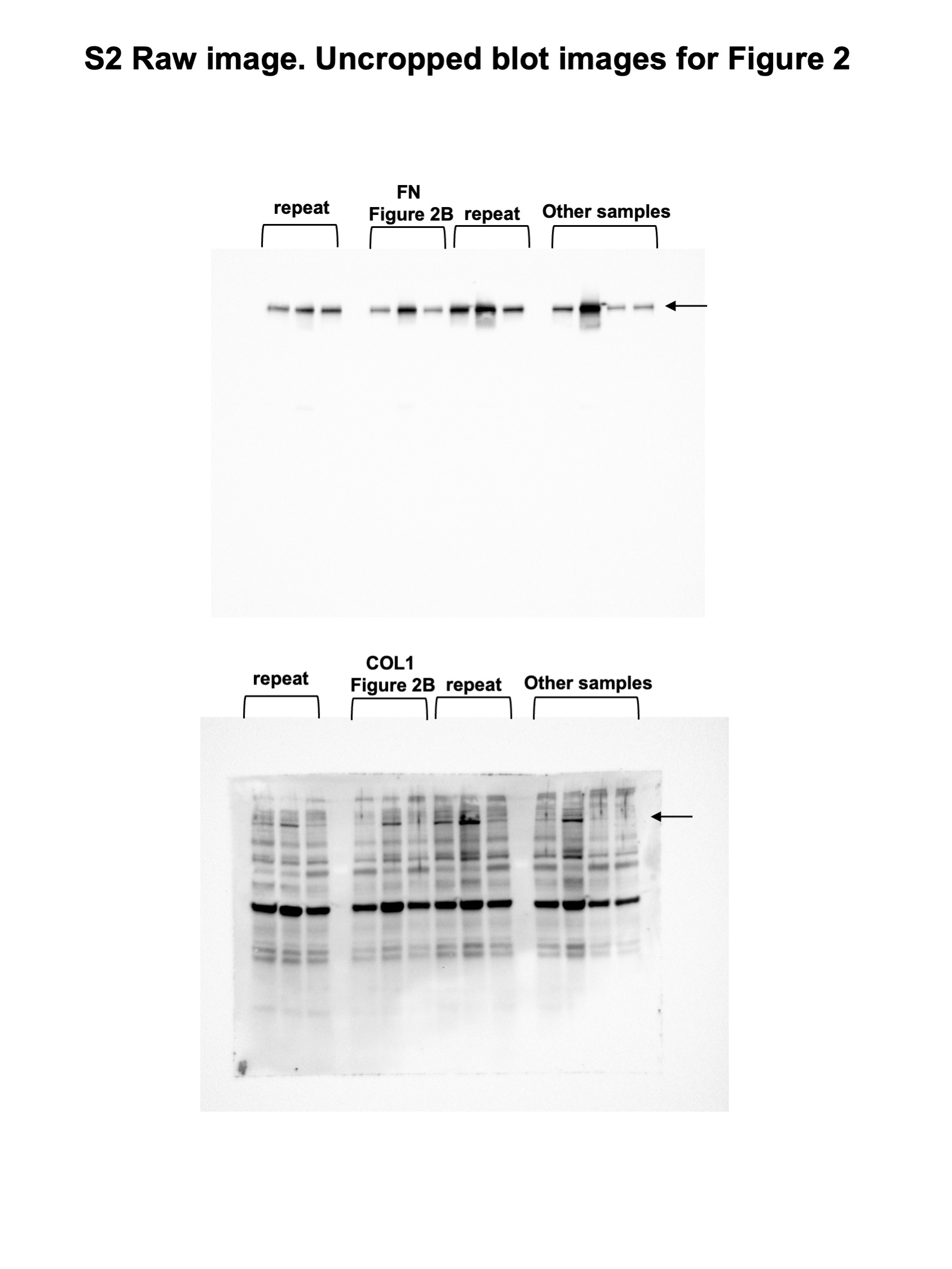
**

**
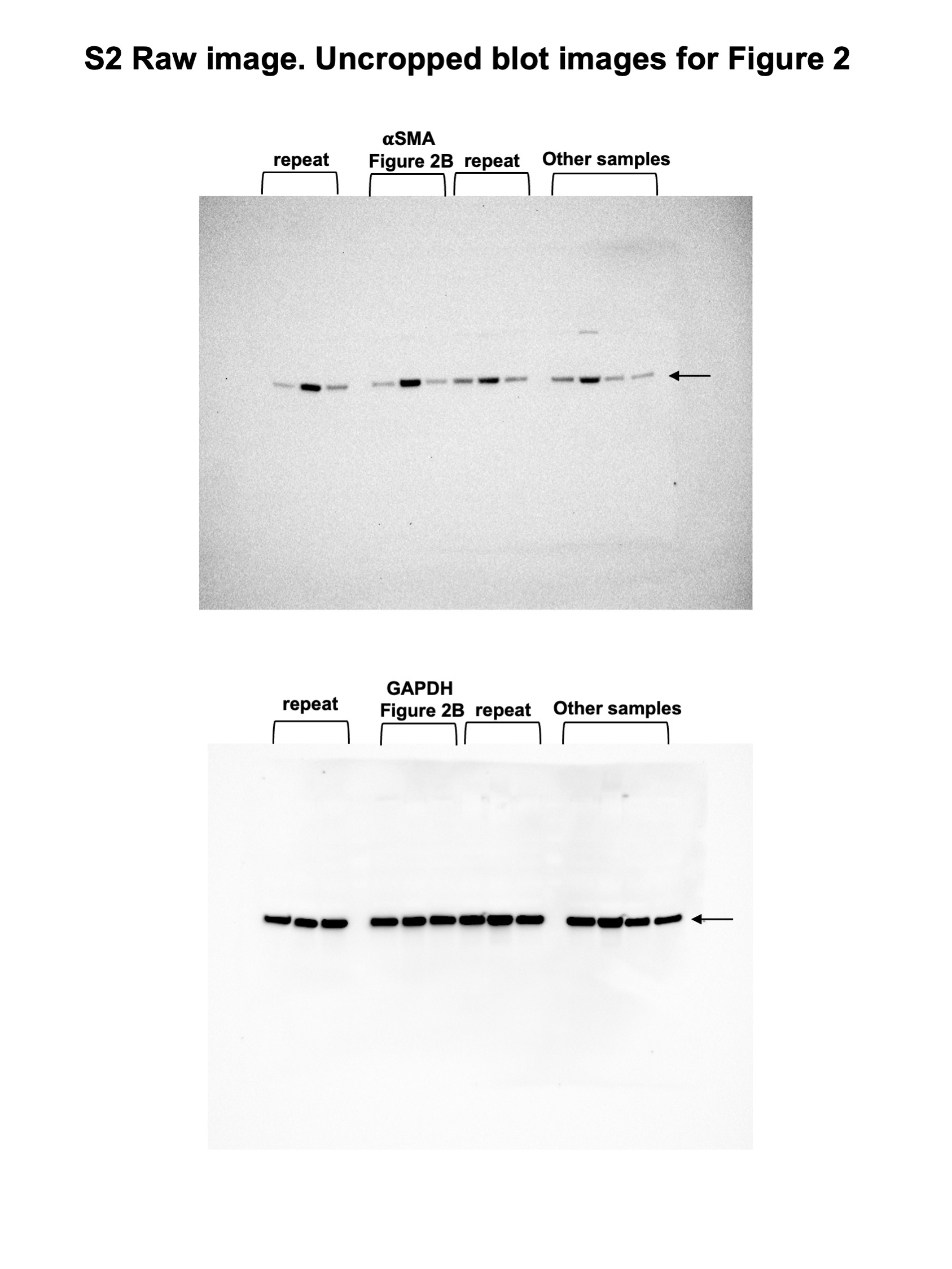
**

**
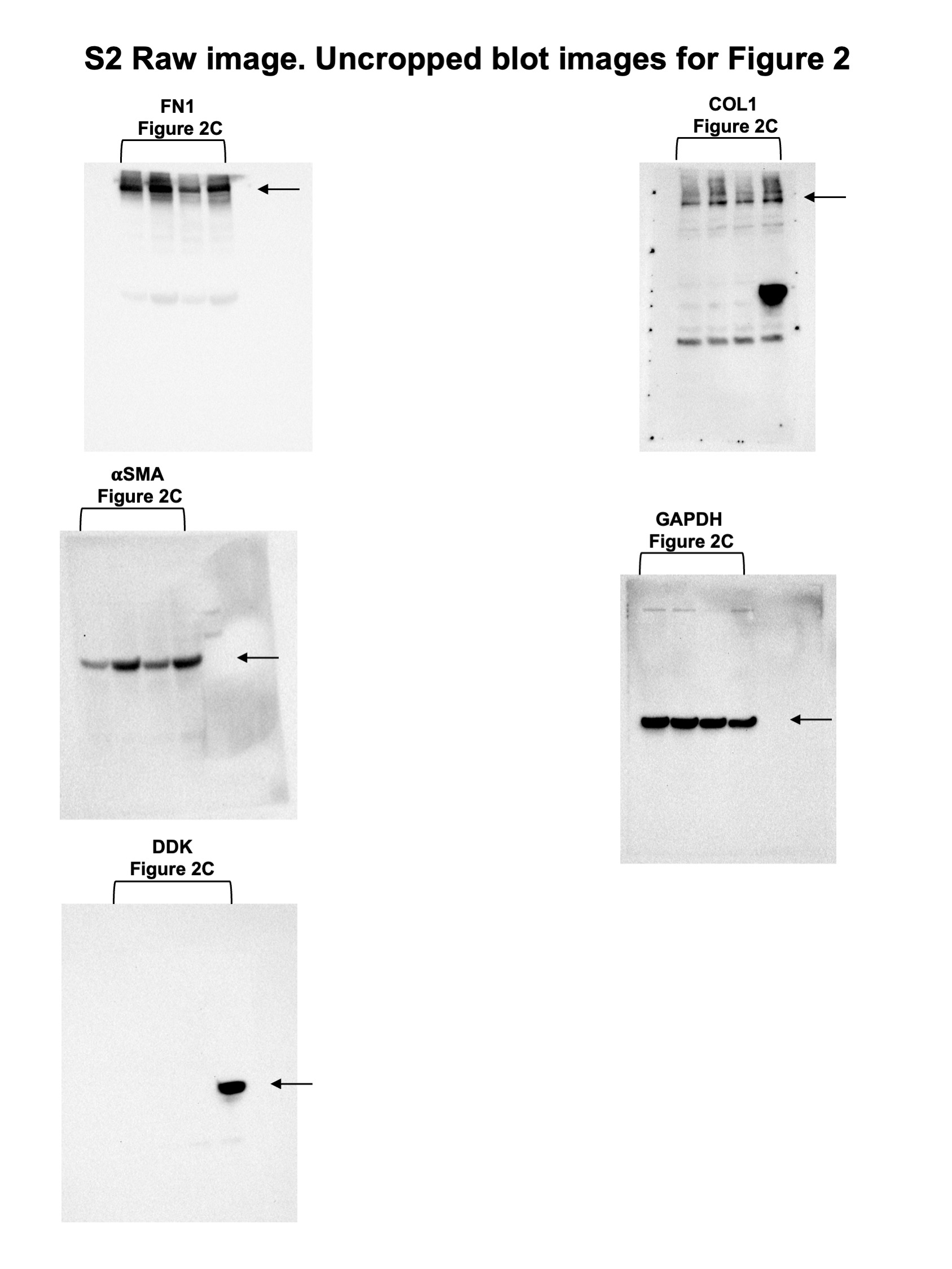
**

**
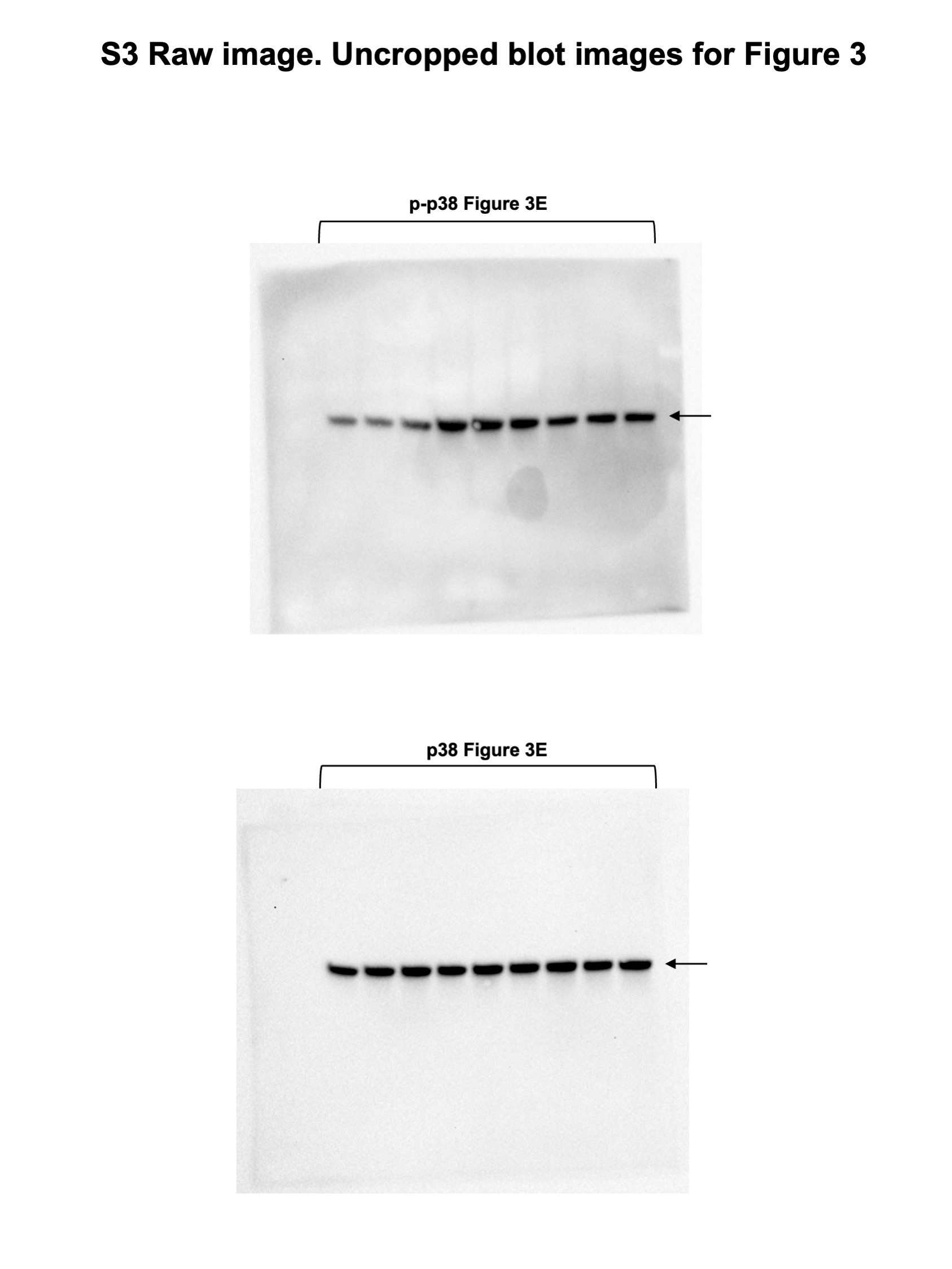
**

**
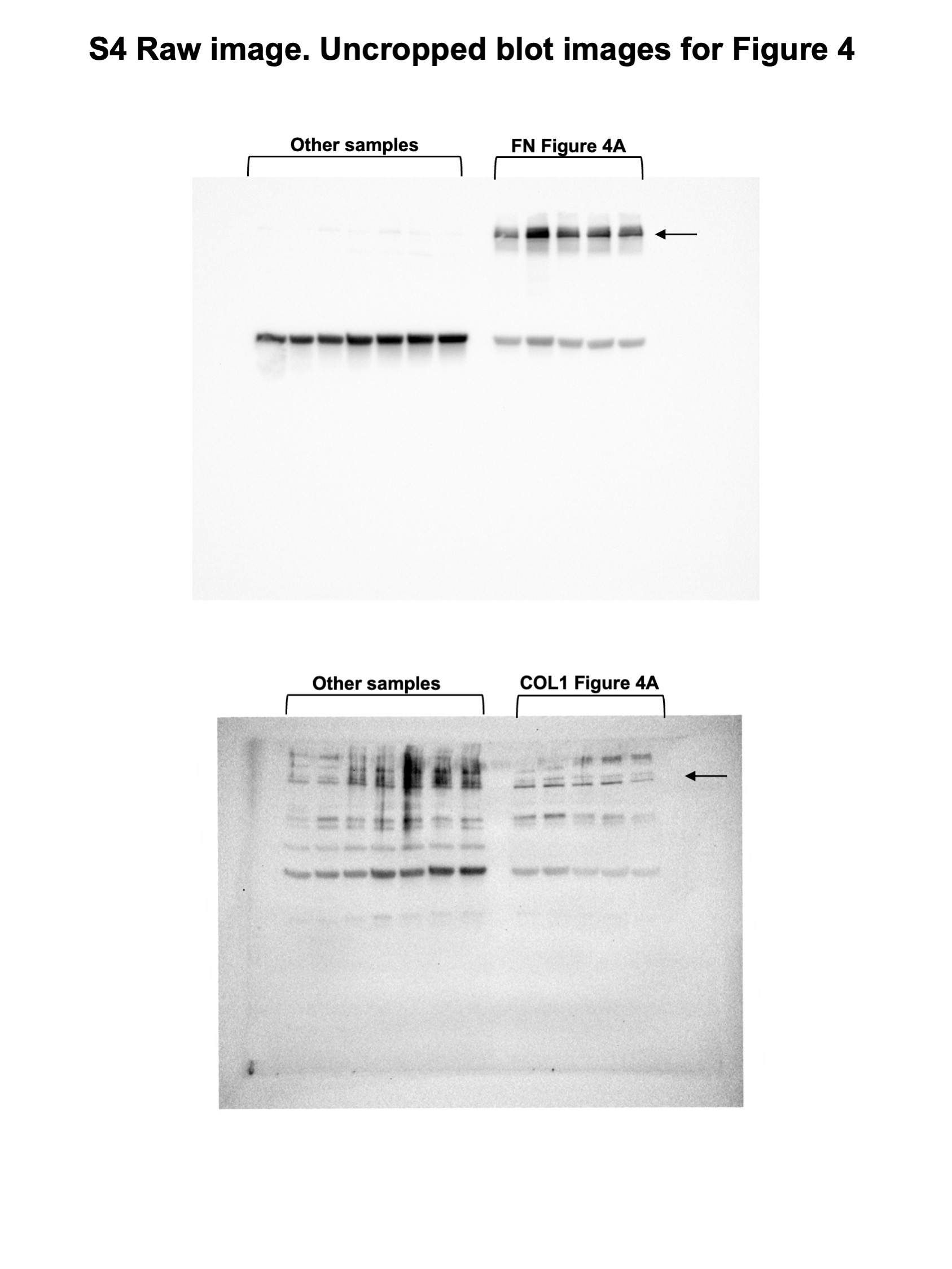
**

**
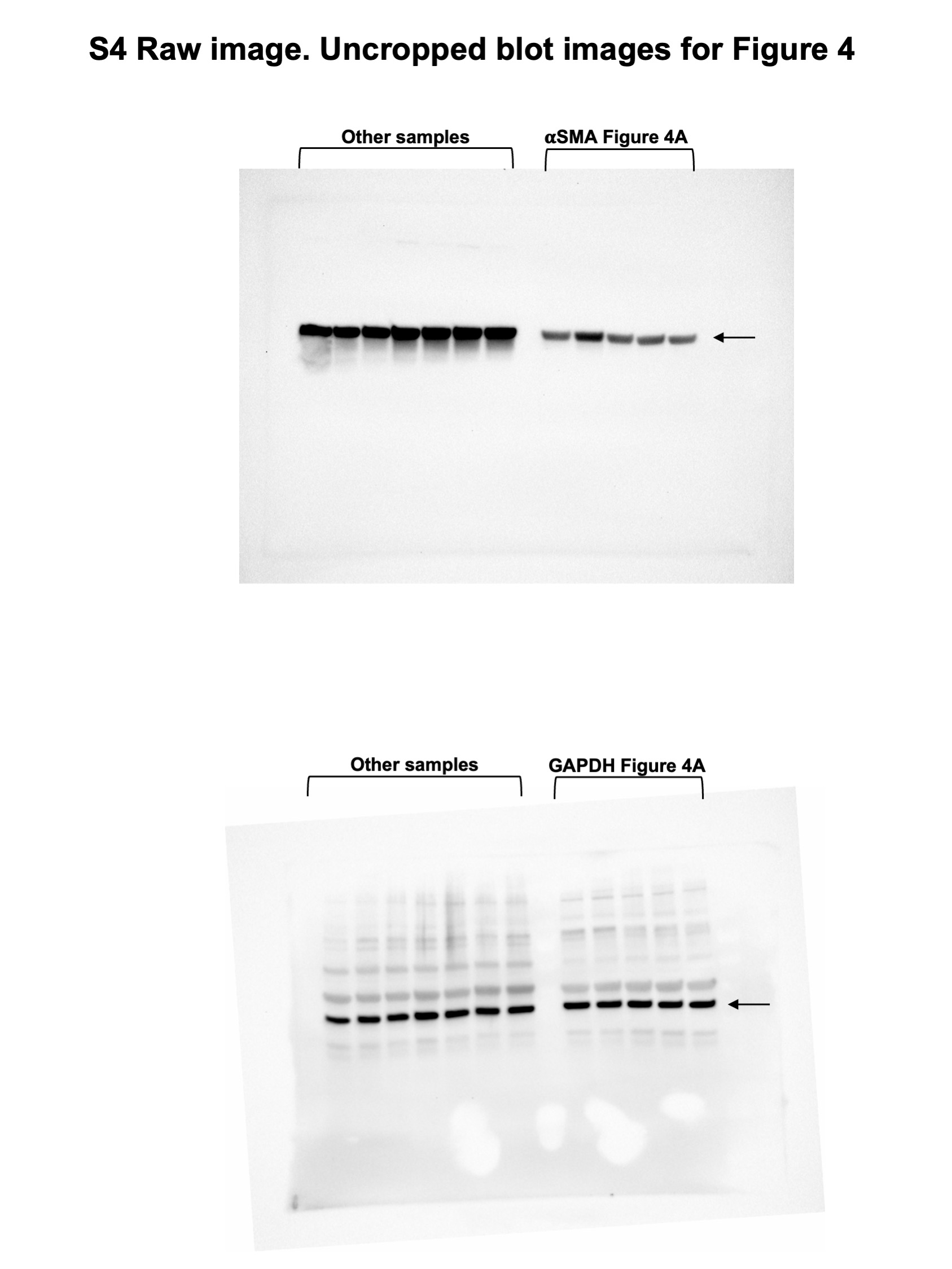
**

**
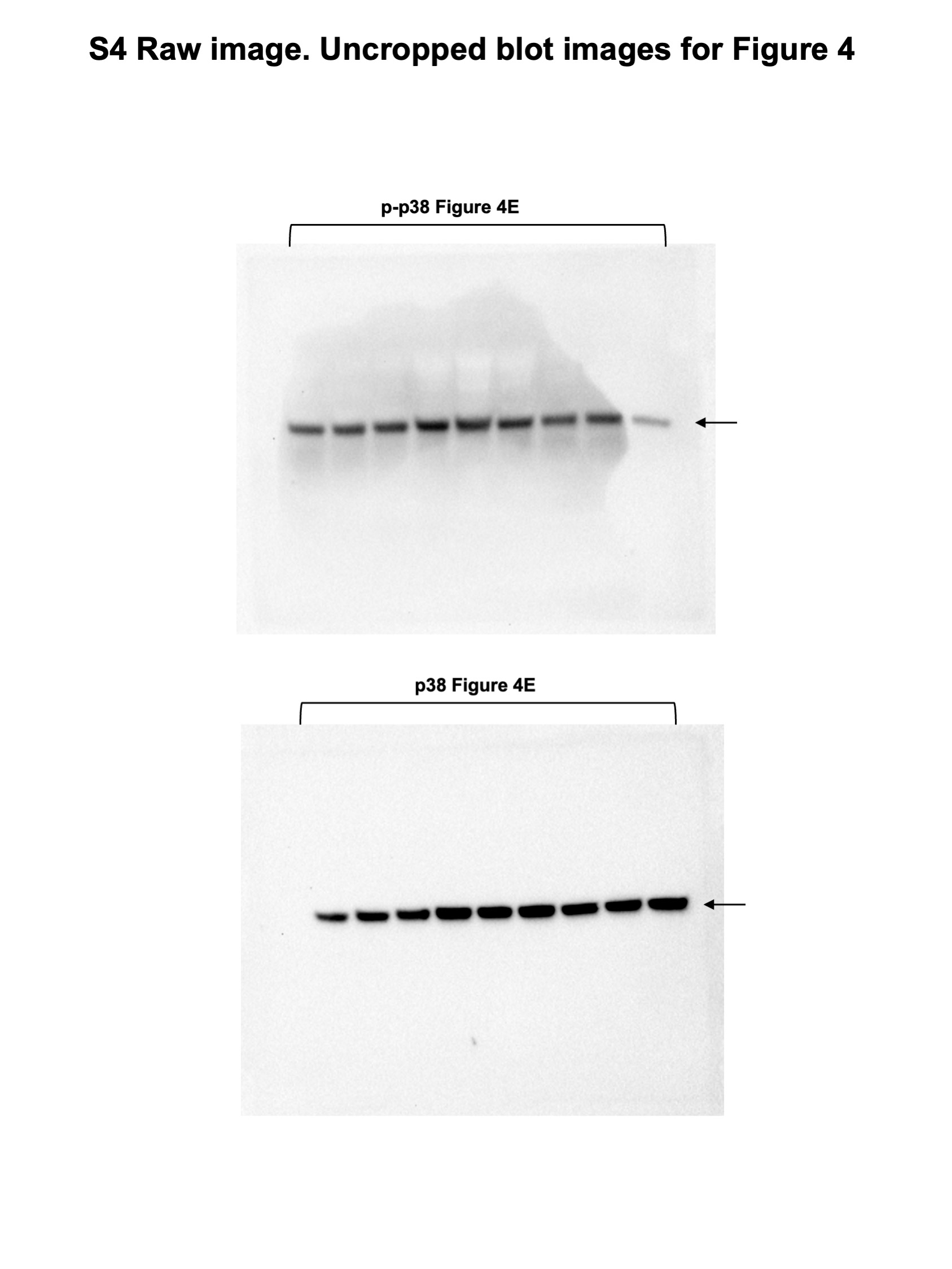
**

**
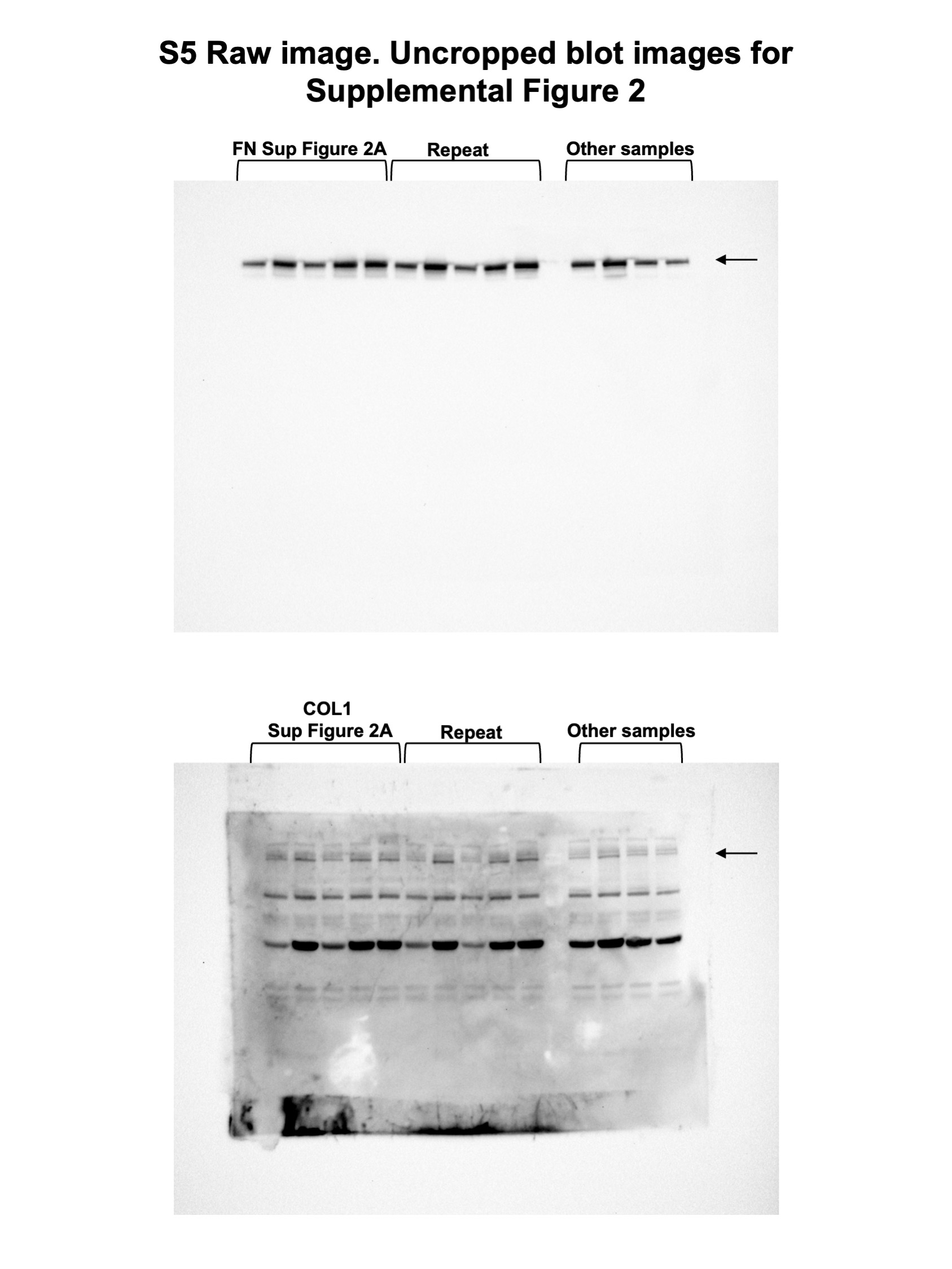
**

**
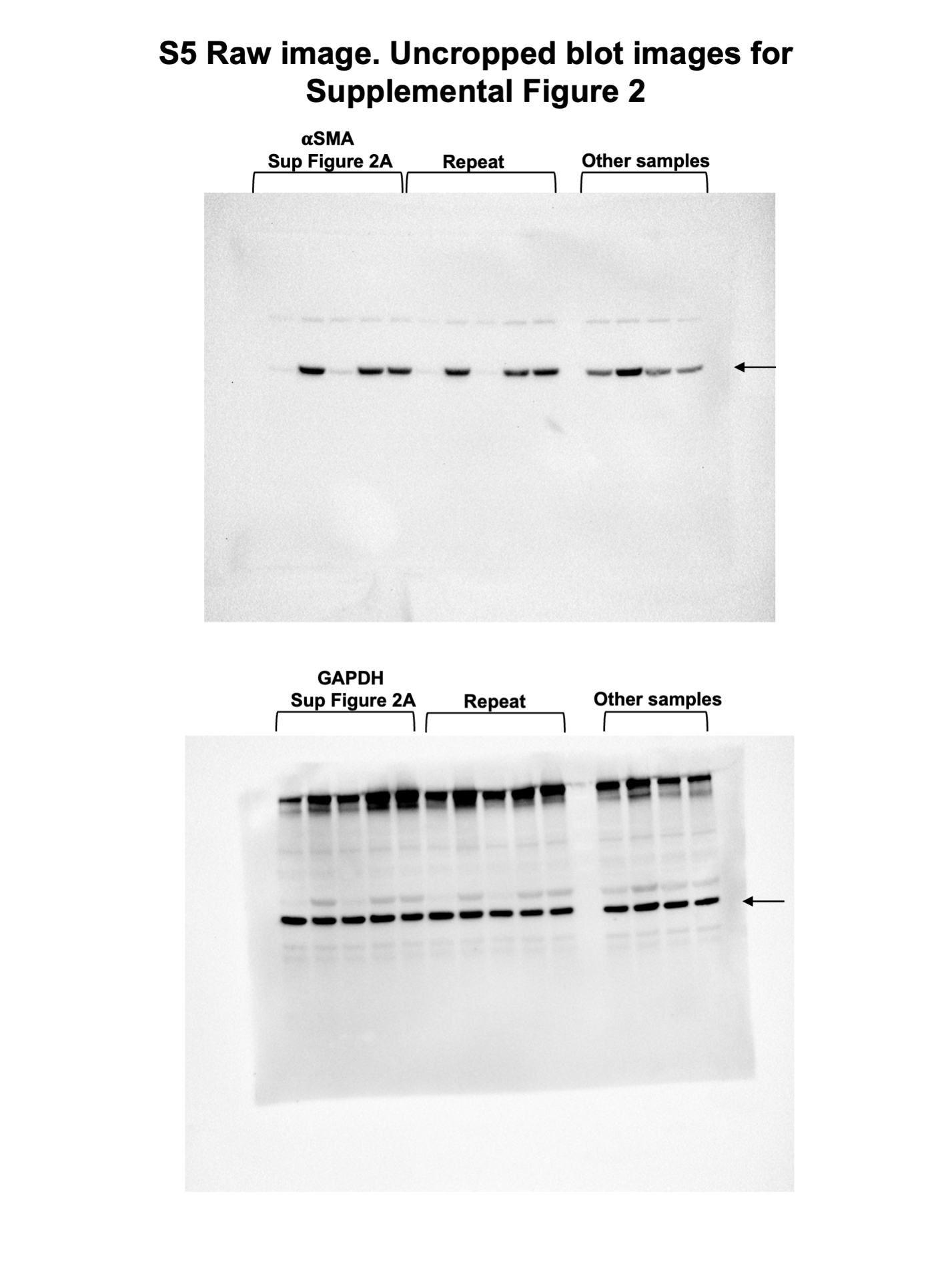
**

**
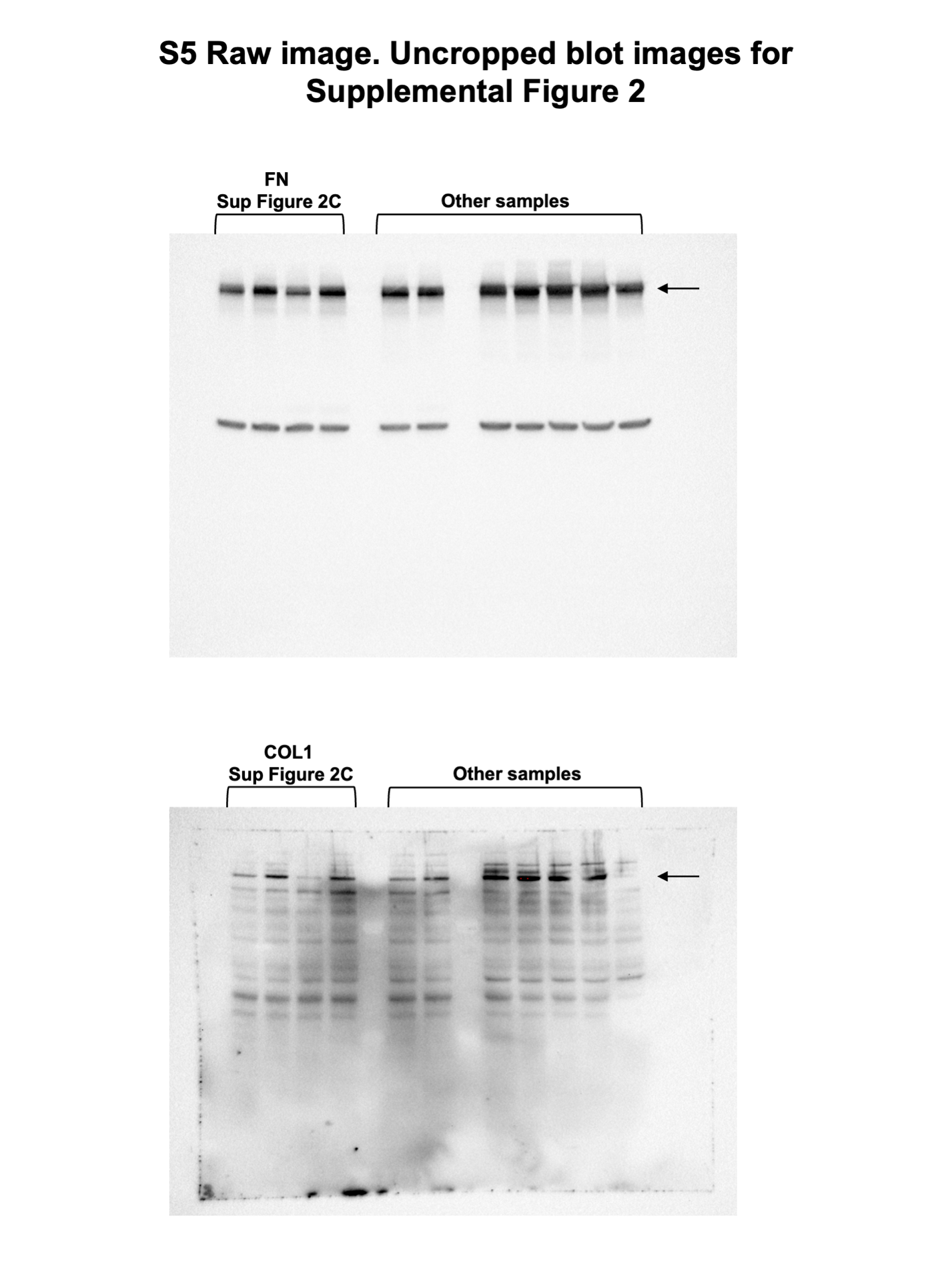
**

**
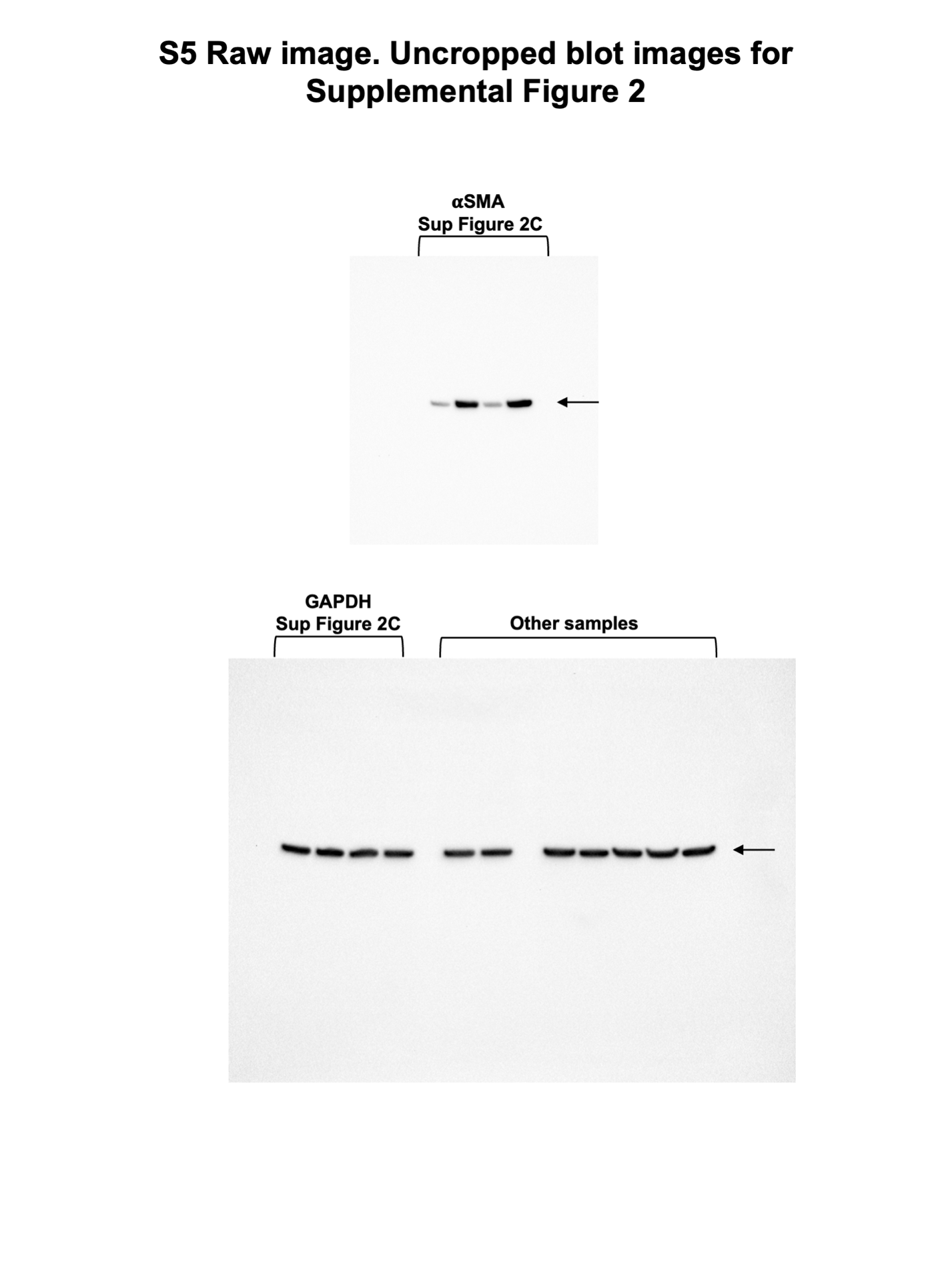
**

**
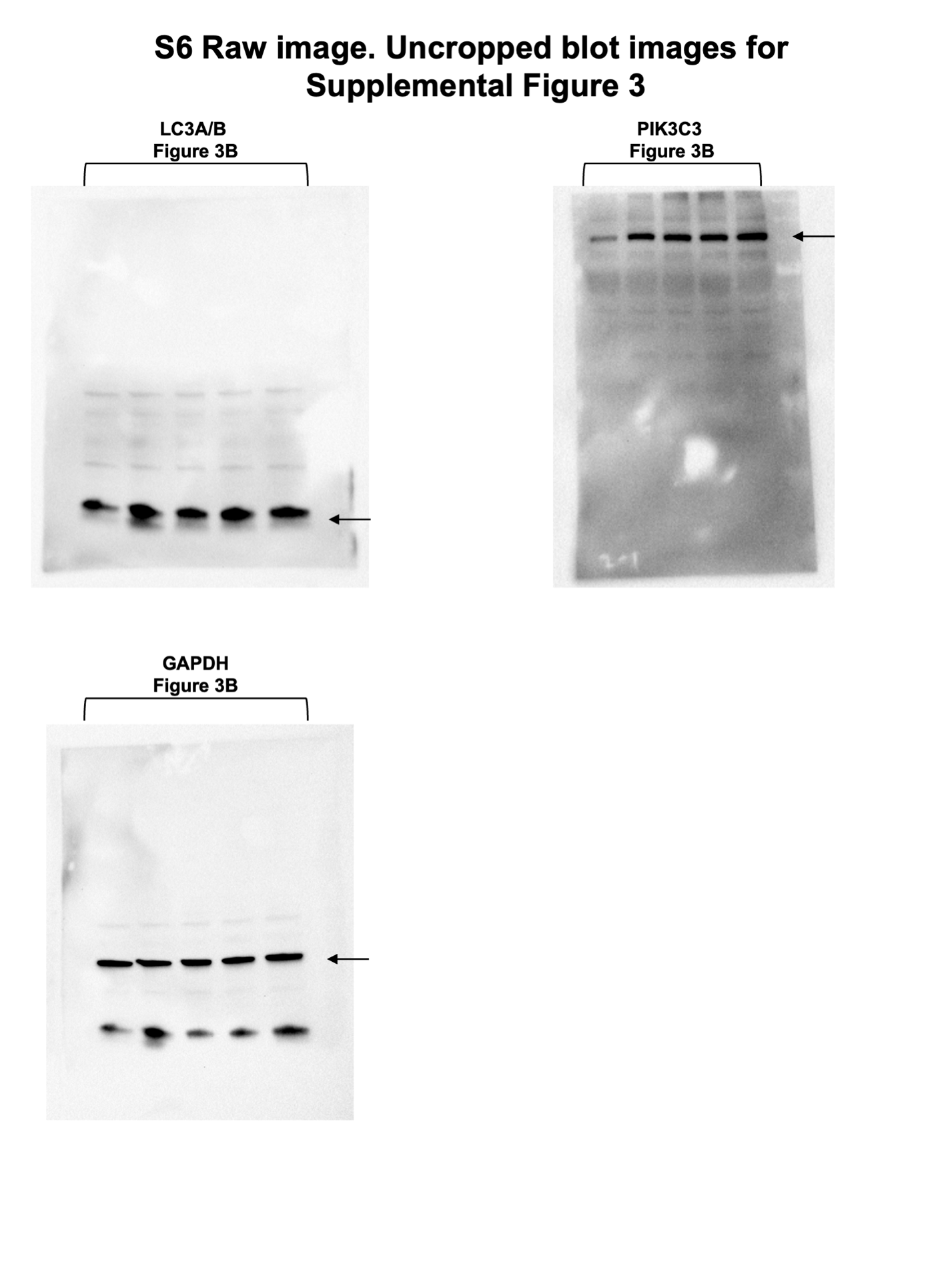
**

**
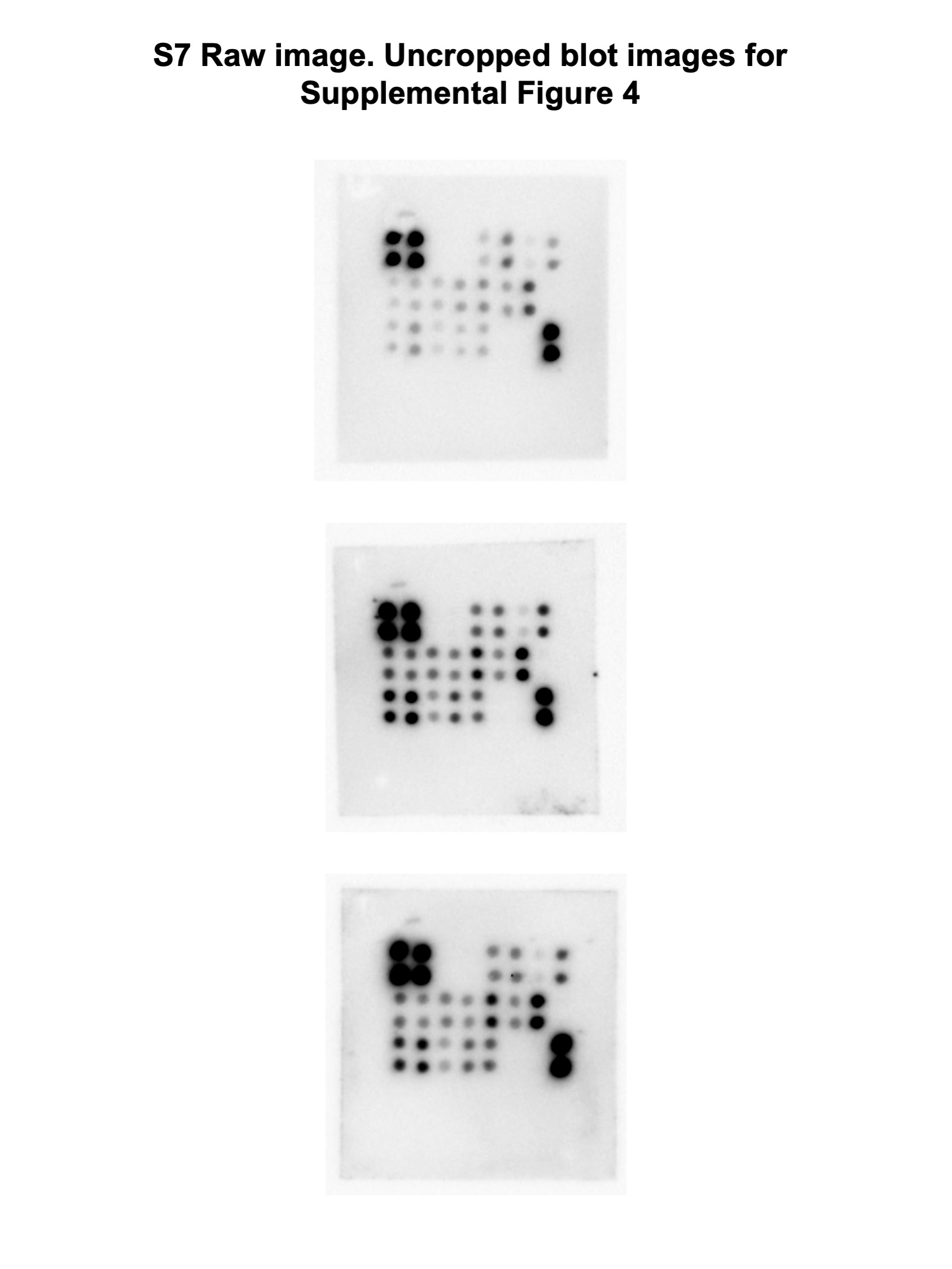
Supporting Information**

**S1 Figure:** Effect of PIK-III on cell viability in human dermal fibroblasts.

**S2 Figure:** Effect of Vps34 inhibitors and its silencing on profibrotic responses in activated human dermal fibroblasts.

**S3 Figure:** Effect of PIK-III, SAR405 and Autophinib on Vps34-mediated autophagy.

**S4 Figure:** Effect of PIK-III on the activation of different signaling proteins in activated human dermal fibroblasts.

**S5 Figure:** Representative H&E staining images of the mouse lungs.

**S6 Table:** Table of primers and primary antibodies used in the current manuscript.

**S1 Raw image:** Uncropped blot images for FN, COL1, αSMA and GAPDH used in figure 1A.

**S2 Raw image:** Uncropped blot images for p-p38, p38 used in figure 2A, FN, COL1, αSMA and GAPDH used in figure 2B and FN, COL1, αSMA, GAPDH and DDK used in figure 2C.

**S3 Raw image:** Uncropped blot images for p-p38, p38 used in figure 3E.

**S4 Raw image:** Uncropped blot images for FN, COL1, αSMA and GAPDH used in figure 4A, p-p38, p38 used in figure 4E.

**S5 Raw image:** Uncropped blot images for FN, COL1, αSMA and GAPDH used in Supplemental Figure 2A and FN, COL1, αSMA and GAPDH used in Supplemental Figure 2C.

**S6 Raw image:** Uncropped blot images for LC3A/B, PIK3C3 and GAPDH used in Supplemental Figure 3B.

**S7 Raw image:** Uncropped blot images for antibody phospho array used in Supplemental Figure 4.
